# ﻿Stage-specific RNA regulomes of *Trichophyton mentagrophytes*: mRNA-lncRNA-miRNA interplay in spore-hypha transition

**DOI:** 10.3897/imafungus.16.166433

**Published:** 2025-11-05

**Authors:** Wudian Xiao, Zhaodan Wu, Jia Zhang, Jun Wan, Ruihuan Zhang, Xinyi Xiang, Yang Yu, Lu Fu, Kui Yang, Yang Chen, Ziyao Xiao, Ziyu Wang, Lvqin He, Jingcan You, Chunxiang Zhang

**Affiliations:** 1 Basic Medicine Research Innovation Center for cardiometabolic diseases, Ministry of Education, Department of Cardiology, The Affiliated Hospital of Southwest Medical University, Key Laboratory of Medical Electrophysiology, Ministry of Education, Institute of Cardiovascular Research, Nucleic Acid Medicine of Luzhou Key Laboratory, Model Animal and Human Disease Research of Luzhou Key Laboratory, Laboratory Animal Center, School of Basic Medical Sciences, School of Clinical Medicine, School of Public Health, Southwest Medical University, Luzhou, 646000, China Southwest Medical University Luzhou China; 2 Department of Orthodontics, State Key Laboratory of Oral Diseases, National Clinical Research Center for Oral Diseases, West China Hospital of Stomatology, Sichuan University, Chengdu, China Sichuan University Chengdu China; 3 College of Intelligent Manufacturing, Changchun Sci-Tech University, Changchun, 130600, China Changchun Sci-Tech University Changchun China; 4 Department of Respiratory and Critical Care Medicine, Luzhou People's Hospital, Luzhou, China Luzhou People's Hospital Luzhou China

**Keywords:** Hyphae, lncRNAs, miRNAs, mRNAs, RNA-sequencing, Spores, *Trichophyton mentagrophytes*

## Abstract

**Background**: As a globally distributed dermatophyte, *Trichophyton mentagrophytes* (*T. mentagrophytes*) causes diverse dermatophytoses in humans and animals. Long non-coding RNAs (lncRNAs) and microRNAs (miRNAs), which serve as critical regulators of diverse biological processes, have been extensively characterized in numerous fungal species. However, the role of mRNAs, lncRNAs, and miRNAs during *T. mentagrophytes* germination remains unexplored.

**Objectives**: In this study, the molecular mechanisms involved in the germination of *T. mentagrophytes* were systematically investigated.

**Methods**: RNA-sequencing technology, small RNA-sequencing technology, related bioinformatics methods, and qRT-PCR were used to systematically characterize the expression profiles of mRNAs, miRNAs, and lncRNAs in *T. mentagrophytes* spores and hyphae, and analyze the regulatory mechanisms of mRNAs, miRNAs, and lncRNAs during *T. mentagrophytes* germination.

**Results**: In our study, RNA-sequencing was performed to identify mRNAs, lncRNAs, and miRNAs in spores and hyphae of *T. mentagrophytes*. A total of 3,193 differentially expressed mRNAs, 409 differentially expressed lncRNAs, and 119 differentially expressed miRNAs were identified, with qRT-PCR subsequently used to verify the dependability of the sequencing data. In addition, an mRNA-lncRNA-miRNA regulatory network containing 2,672 mRNAs, 107 miRNAs, and 329 lncRNAs was constructed. Gene Ontology, Kyoto Encyclopedia of Genes and Genomes, and Gene Set Enrichment Analysis suggested that mRNAs, lncRNAs, and miRNAs may play important roles during spore germination, potentially participating in fundamental biosynthetic, cell wall remodelling, cell cycle regulation, cytoskeletal reorganization, epigenetic regulation, and metabolic processes.

**Conclusion**: Our study revealed the characteristics of mRNAs, lncRNAs, and miRNAs in *T. mentagrophytes* using transcriptomic methods, and set the stage for future pathogenicity studies and antifungal drug development for *T. mentagrophytes*.

## ﻿Introduction

*Trichophyton mentagrophytes* (*T. mentagrophytes*) is a globally distributed zoonotic dermatophyte that infects keratinized tissues (skin, nails, hair follicles), causing persistent dermatophytoses in humans and diverse mammalian hosts. Clinical manifestations include tinea manuum, onychomycosis, tinea capitis, and tinea pedis (Weitzman and Summerbell 1995; Chermette et al. 2008). The recalcitrance of these infections characterized by high recurrence rates and escalating antifungal resistance due to therapeutic overuse, establishes *T. mentagrophytes* as a critical public health threat (Ebert et al. 2020).

The spore-to-hypha transition constitutes a pivotal virulence determinant. During germination, spores adhere to the stratum corneum via extracellular ligands, develop germ tubes, and secrete keratinolytic enzymes (keratinases, lipases, phospholipases) that facilitate tissue invasion (Duek et al. 2004; Elavarashi et al. 2017). Notably, while scanning electron microscopy has delineated morphological events, the transcriptomic regulatory architecture—particularly dynamic interactions among mRNAs, lncRNAs, and miRNAs, remains unexplored in *T. mentagrophytes* (Duek et al. 2004).

Advancements in RNA sequencing (RNA-seq) enable genome-scale profiling of transcriptional networks (Hrdlickova et al. 2017), as demonstrated by antifungal target identification in *Trichophyton rubrum* (Galvão-Rocha et al. 2023). Non-coding RNAs that are transcribed from non-coding regions of the genome, orchestrate key fungal adaptations. Long non-coding RNAs (lncRNAs; >200 nt) modulate cell wall biogenesis, transcriptional regulation (e.g., GAL-mediated R-loop formation in yeast), and stress responses (Cloutier et al. 2016; Hombach and Kretz 2016; Novačić et al. 2020; Shuman 2020). MicroRNAs (miRNAs; 19–25 nt) guide RNA-induced silencing complexes (RISCs) to target mRNAs via 3’/5’ UTR binding, post-transcriptionally regulating developmental transitions (Iwakawa and Tomari 2022), as evidenced by stage-specific miRNAs in *Trichophyton rubrum* and iron adaptation in *Paracoccidioides brasiliensis* (Wang et al. 2018; de Curcio et al. 2021). Despite documented roles of lncRNAs and miRNAs in fungal morphogenesis, no integrated analysis of the tripartite RNA regulome (mRNA-lncRNA-miRNA) exists for *T. mentagrophytes* germination (Lau et al. 2020; Lai et al. 2023).

Recently, we reported a transcriptome analysis of circRNAs in *T. mentagrophytes* during spore germination (Zhang et al. 2023). That work provided the first insights into RNA-based regulation of fungal morphogenesis in dermatophytes, but was inherently restricted to a single class of non-coding RNAs. To achieve a more comprehensive understanding, the present study integrates expression profiles of mRNAs, lncRNAs, and miRNAs into a unified regulatory framework. This integrated approach allows us to not only catalogue diverse RNA species but also to construct candidate competing endogenous RNA (ceRNA) networks, thereby exploring the multilayered control underlying the critical spore-to-hypha transition.

## ﻿Materials and methods

### ﻿Fungal culture and sample collection

The *T. mentagrophytes* wild-type strain ATCC MYA-4439 was provided by BeNa Culture Collection (BNCC, Beijing, China). *T. mentagrophytes* strain was cultured and maintained on potato dextrose agar medium (BD, Sparks, MD, USA) for 14 days at 28 °C to harvest spores (Wang et al. 2022; Zang et al. 2024). Spores on the mycelium surface were rinsed using 5 mL of sterile distilled water and passed sequentially through three cell filters with pore sizes of 0.05 mm, 0.026 mm, and 0.01 mm (Zhang et al. 2023). In order to collect hyphae, spores were cultured and maintained in yeast extract peptone dextrose medium solution shaken on a THZ-300 shaker (Yiheng Scientific Instrument Co., Shanghai, China) at 200 rpm for 5 days at 28 °C. The hyphae were then filtered through gauze, followed by washing with sterile distilled water to collect pure hyphae.

### ﻿Germination analysis of *T. mentagrophytes* spores

For germination analysis, 20 mL of 1 × 10^5^ spores/mL *T. mentagrophytes* spore suspension was inoculated into each of a series of 250 mL erlenmeyer flasks containing 80–100 mL of yeast extract peptone dextrose medium, and immediately incubated at 28 °C with constant shaking (200 rpm) (Yiheng Scientific Instrument Co., Shanghai, China). Samples at the time-points 0, 6, 12, and 18 hr post-inoculation were taken directly from the medium and subjected to morphological observation using a motic AE2000 microscope (Motic, Fujian, China).

### ﻿mRNA-lncRNA isolation, ribosomal RNA-depleted strand-specific library construction, and sequencing

After total RNA was extracted from the three spore (S, n = 3) and hyphae (H, n = 3) samples using TRIzol^®^ reagent (Invitrogen, Carlsbad, CA, USA) according to the manufacturer’s instructions, ribosomal RNA was removed to retain both coding and non-coding RNAs. The remaining RNA was enriched using SPRI beads, fragmented at high temperature, and reverse-transcribed into double-stranded cDNA. The cDNA was then end-repaired, A-tailed, and ligated to adapters, followed by PCR amplification. Amplified products were purified with VAHTS DNA Clean Beads and sequenced on an Illumina NovaSeq X Plus by Gene Denovo Biotechnology Co. (Guangzhou, China).

### ﻿miRNA isolation, library construction, and sequencing

Total RNA of spores (S, n = 3) and hyphae (H, n = 3) was extracted with the TRIzol^®^ reagent, followed by enrichment of RNA molecules in the 18–30nt size range by polyacrylamide gel electrophoresis (PAGE). The library construction involved sequential ligation of adapters. After the 3’ adapters were ligated, a size-selection step was performed to enrich RNAs between 36–44 nucleotides. The 5’ adapters were then ligated to these size-selected molecules. The ligation products were then reverse transcribed by PCR amplification, and the 140–160 bp size PCR products were enriched to generate a cDNA library and sequenced using Illumina NovaSeq X Plus by Gene Denovo Biotechnology Co. (Guangzhou, China).

### ﻿Analysis of differentially expressed mRNA

Initially, low-quality reads were filtered from the raw data using fastp (version 0.18.0) to obtain high-quality clean reads (Chen et al. 2018). Reads mapped to the ribosome database were then removed using Bowtie2 (version 2.2.8) (Langmead and Salzberg 2012). Paired-end clean reads were mapped to the *T. mentagrophytes* genome (GSA: CRA028745) using HISAT2 (version 2.2.1), and transcripts were reconstructed using Stringtie (version 2.2.3) (Trapnell et al. 2010; Kim et al. 2015; Pertea et al. 2015). All of the reconstructed transcripts were aligned to reference genome and were divided into twelve categories by using gffcompare. We defined transcripts with one of the class codes ‘u, i, j, x, c, e or o’ as novel transcripts. We used the following parameters to identify reliable novel genes: transcripts longer than 200 bp, and had to have more than two exons. A FPKM (fragment per kilobase of transcript per million mapped reads) value was calculated using the RSEM software primarily for assessing gene expression levels and facilitating sample comparisons (Li and Dewey 2011). Correlation analysis was conducted using R to assess the reliability and operational stability of the experimental replicates. The correlation coefficient between two samples was calculated, with values closer to 1 indicating stronger reproducibility. Principal component analysis (PCA) was performed using the R package gmodels (http://www.rproject.org/) to reveal relationships between samples. To ensure analytical rigor, differential expression analysis was performed using DESeq2, which utilizes raw read counts and employs a negative binomial model to identify significantly differentially expressed genes (Love et al. 2014). Genes with a false discovery rate (FDR) < 0.05 and a fold change > 2 were considered differentially expressed.

### ﻿Identification and analysis of lncRNAs

All reconstructed transcripts were mapped to the *T. mentagrophytes* genome and classified into twelve categories using Cuffcompare before selecting transcripts longer than 200 bp and with an exon number greater than two. By requiring more than one exon, our pipeline systematically excludes single-exon lncRNAs. The protein coding potential of the new transcripts was assessed with CNCI (version 2), CPC (version 0.9-r2) and FEELNC (version v0.2) using the default parameters (Kong et al. 2007; Sun et al. 2013; Wucher et al. 2017). Transcripts consistently predicted as non-protein-coding by all three tools were classified as lncRNAs. Potential lncRNAs were classified into five categories based on their position with respect to protein coding genes: intergenic lncRNAs, bidirectional lncRNAs, intronic lncRNAs, *antisense*lncRNAs, and sense-overlapping lncRNAs. The lncRNA target mRNAs were identified by performing *antisense*, *trans*- and *cis*-regulation lncRNA analyses with RNAplex (version 0.2) (Tafer and Hofacker 2008). The FPKM values, correlation coefficients, PCA, and differentially expressed lncRNAs were assessed as for mRNA (see section 2.5 above).

### ﻿miRNA identification and analysis

Reads containing more than one low quality (Q-value ≤ 20) base or unknown nucleotides (N), reads without 3’ adapters, reads containing 5’ adapters, reads containing 3’ and 5’ adapters but no small RNA fragment between them, reads containing poly (A) in the small RNA fragment, and reads shorter than 18 nt (not including adapters) were removed from the raw data. The resulting clean tags were aligned to the GenBank database (Release 209.0) and Rfam database (Release 14.10) to remove rRNA, scRNA, snoRNA, snRNA and tRNA. All clean tags were also aligned to the reference genome (GSA: CRA028745) to remove fragments and repetitive sequences that mapped to exons or introns. Clean tags were aligned to the fungal miRBase database (Release 22) to identify known miRNAs using Bowtie (version 1.1.2). Subsequently, the remaining unaligned tags were compared against miRNA sequences from other species within miRBase, applying a filter that required alignment to precursor sequences while excluding matches beyond the 2 bp extensions of the mature miRNA. The novel miRNA candidates were identified by mirDeep2 based on their genome positions and predicted hairpin structures, after excluding all tags matching known miRNAs, miRNA editing variants, mRNA degradation fragments, repetitive regions, and other non-coding RNAs (including rRNA, scRNA, snoRNA, snRNA, and tRNA) (Mackowiak 2011). miRNA target mRNAs were identified by patmatch (Version 1.2). The transcripts per million (TPM) value was calculated with the following formula: TPM = Actual miRNA counts/Total counts of clean tags × 10^6^. Correlation coefficients and PCA were assessed as for mRNA (see section 2.5 above). We identified miRNAs with a fold change > 2 and *P* < 0.05 in a comparison as significant differentially expressed miRNAs.

### ﻿Enrichment analysis

Gene ontology (GO) enrichment analysis and Kyoto Encyclopedia of Genes and Genomes (KEGG) pathway analysis were performed on differentially expressed mRNAs, lncRNAs target mRNAs, miRNAs target mRNAs and ceRNA target mRNAs. For GO enrichment analysis, all differentially expressed genes (DEGs) were mapped to GO terms (http://www.geneontology.org/). The number of genes associated with each term was calculated. GO terms that were significantly enriched in the DEGs compared to the expressed gene universe were identified using a hypergeometric test. Pathway enrichment analysis identified significantly enriched metabolic pathways or signal transduction pathways in DEGs comparing with the expressed gene universe as background gene sets. GO terms and KEGG pathways with FDR ≤ 0.05 were considered significantly enriched. Subsequently, GO enrichment analysis and KEGG pathway analysis were performed on a set of genes by using the Gene Set Enrichment Analysis (GSEA) (Subramanian et al. 2005). Gene sets with |NES| > 1, NOM *p-val* < 0.05, and *FDR q-val* < 0.25 were considered differentially expressed.

### ﻿Orthology-inferred Protein-Protein interaction (PPI)

PPI analysis was performed using the STRING v10 database and visualized in Cytoscape (v3.7.1) (Szklarczyk et al. 2015). Since STRING does not contain specific PPI data for *T. mentagrophytes*, an orthology-based approach was employed. Protein sequences of *T. mentagrophytes* were mapped to the *Saccharomyces cerevisiae* reference proteome using reciprocal best BLAST hit orthology inference. The resulting orthology-inferred PPI network was analyzed to identify putative functional modules and hub proteins. Putative hub genes were identified by intersecting the top-ranked candidates from the cytoHubba plugin (evaluated by Degree, Edge Percolated Component (EPC), Eccentricity, and Maximum Neighborhood Component algorithms (MNC)) with key modules derived from Molecular Complex Detection (MCODE) analysis in Cytoscape. The resulting protein-protein interaction network was constructed based on these high-confidence hub genes (Shannon et al. 2003).

### ﻿Construction and analysis of candidate lncRNA – miRNA – mRNA network (ceRNA)

CeRNA refers to the pool of transcripts (such as mRNA and lncRNA) that can competitively sequester miRNA activity. Based on the same miRNA response elements (MREs), candidate ceRNA networks between lncRNAs, mRNAs, and miRNA were constructed by assembling differentially expressed mRNAs, lncRNAs and miRNAs. Depending on differentially expressed mRNAs, lncRNAs, and miRNAs, expression correlation between mRNA-miRNA or lncRNA-miRNA with a Spearman Rank correlation coefficient (SCC) ≤ -0.7 were identified as negatively co-expressed lncRNA-miRNA pairs or mRNA-miRNA pairs. Expression correlation between lncRNA-mRNA with a Pearson correlation coefficient (PCC) > 0.9 were selected as co-expressed lncRNA-mRNA pairs. The potential lncRNA-mRNA-miRNA pairs with *p*-values less than 0.05 (without multiple-testing correction) in the above analysis were screened by the hypergeometric distribution test to obtain the final ceRNA pairs. All co-expressed competing triplets were then assembled to construct ceRNA and visualized with Cytoscape software (v3.6.0). The number of co-expressed targeted miRNAs was defined as the RNA connectivity to identify hub genes.

### ﻿qRT-PCR validation

To validate the RNA-seq results, quantitative real-time polymerase chain reaction (qRT-PCR) was conducted for four randomly selected differentially expressed mRNAs, four differentially expressed lncRNAs, and six differentially expressed miRNAs. Total RNA was extracted from spores and hyphae, followed by reverse transcription to cDNA with ReverRra Ace qPCR RT Master Mix (Toyobo, Osaka, Japan). Next, qRT-PCR was conducted with PowerUpTM SYBRTM Green Master Mix (Applied Biosystems, Thermo Fisher Scientific, Waltham, MA, USA). Briefly, the reaction volume contained 5 μL of SYBR Green Master Mix, 1 μL of 10 μM forward and reverse primers, 1 μL of template cDNA, and 3 μL of dH_2_O in a final volume of 10 μL. The reactions were performed on a QuantStudio 5 PCR System (Applied Biosystems, Thermo Fisher Scientific) as follows: 50 °C for 2 min and 95 °C for 2 min, followed by 40 cycles of 95 °C for 1 s and 60 °C for 30 s. A melt curve analysis was performed at 95 °C for 1 s, 60 °C for 20 s, and 95 °C for 1 s. No-template controls and no-reverse transcription controls were included in every qPCR run to monitor contamination. None of these controls showed specific amplification, confirming the specificity and reliability of the experimental results. The amplification efficiency of all qPCR primers was validated via standard curves constructed from serially diluted cDNA templates (typically 2-fold or 10-fold dilutions). Amplification efficiency (E) was calculated using the formula E = (10^(-1/slope) - 1)*100%. The efficiency for all primers used fell within the range of 90%–110%, with R² values for the standard curves greater than 0.98, meeting the recommended criteria of the MIQE guidelines. All reactions produced a single sharp peak in the melt curve analysis, confirming amplification specificity without nonspecific amplification or primer-dimer formation. Three technical replicates were performed for each biological replicate. The Ct values of the three technical replicates for each biological replicate were first averaged to obtain a representative Ct value for that biological replicate. Subsequently, all group-based statistical analyses and data presentations were performed using these consolidated Ct values, each representing one biological replicate. The relative quantification of mRNAs, lncRNAs, and miRNAs was calculated by the 2^-ΔΔCt^ method using *chs* and *U6* as the internal reference (Livak and Schmittgen 2001). The stability of the reference genes chs and U6 was evaluated using the geNorm and NormFinder software. Their expression remained stable across all experimental conditions, with an M value less than 0.5, confirming their suitability as references for data normalization.

### ﻿Statistical analyses

Statistical analyses were performed using SPSS Statistics 20.0 (SPSS Inc., Chicago, IL, USA). Unless otherwise specified, values are expressed as the mean ± standard error of the mean (SEM). *P* < 0.05 was considered statistically significant.

### ﻿Abbreviations

**lncRNAs** Long non-coding RNAs

**miRNAs** Micro RNAs

**ceRNA** Competing endogenous RNA

**cDNA** Complementary DNA

**scRNA** Small cytoplasmic RNA

**rRNA** Ribosomal RNA

**snoRNA** Small nucleolar RNA

**snRNA** Small nuclear RNA

**tRNA** Transfer RNA

**mRNA** Messenger RNA

***T. mentagrophytes*** *Trichophyton mentagrophytes*

**qRT-PCR** Quantitative real-time polymerase chain reaction

**RNA-seq** RNA sequencing

**RISC** RNA-induced Silencing Complex

**UTR** Untranslated region

**PAGE** Polyacrylamide gel electrophoresis

**FPKM** Fragment per kilobase of transcript per million mapped reads

**PCA** Principal component analysis

**FDR** False discovery rate

**TPM** Transcripts per million

**GO** Gene ontology

**KEGG** Kyoto Encyclopedia of Genes and Genomes

**GSEA** Gene Set Enrichment Analysis

**PPI** Protein-Protein interactio

**EPC** Edge Percolated Component

**MNC** Maximum Neighborhood Component

**MCODE** Molecular Complex Detection

**MREs** MiRNA response elements

**SCC** Spearman Rank correlation coefficient

**PCC** Pearson correlation coefficient

**SEM** Standard error of the mea

**MAPK** Mitogen-activated protein kinase

## ﻿Results

### ﻿Germination of *T. mentagrophytes* spores

**Figure 1.**
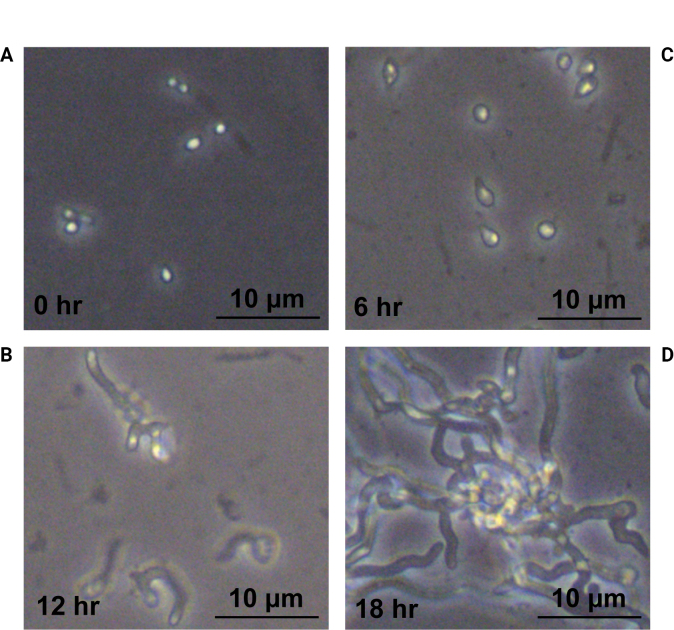
*T. mentagrophytes* observed by optical microscopy during germination. A. 0 h, B. 6 h, C. 12 h, D. 18 h. Scale bar: 10 μm.

Germination of *T. mentagrophytes* spores progressed through two distinct phases: swelling and germ tube formation. Dormant spores displayed a rounded or oval morphology (Fig. 1A). Within 6 hours of incubation, spore swelling commenced, resulting in an approximate doubling of cellular diameter. Concurrently, polarized growth was initiated at one site on the spore surface (Fig. 1B). By 12 hours, the germ tube emergence phase was evident, characterized by the protrusion of a single germ tube (Fig. 1C). Extensive growth of multiple germ tubes, culminating in hyphal formation, was observed by 18 hours (Fig. 1D; Suppl. material 1: fig. S1).

### ﻿Overview of RNA-sequencing

To characterize the expression profiles and co-expression networks of mRNAs, lncRNAs, and miRNAs during *T. mentagrophytes* germination, cDNA and small RNA libraries were constructed and subjected to high-throughput sequencing.

**Figure 2.**
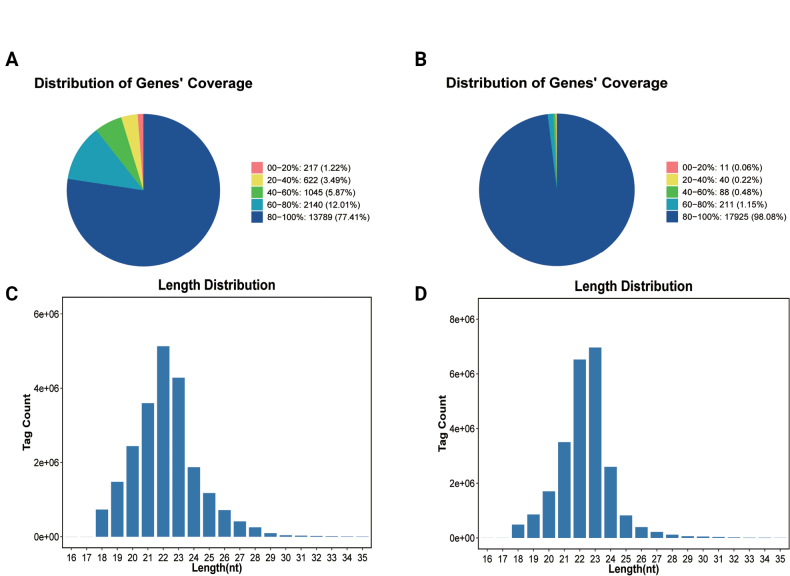
Assessment of sequencing data quality and genome coverage for spore and hyphal samples. A. Distribution of Genes’ Coverage of spore sample represented the percentage of each gene covered by reads. B. Distribution of Genes’ Coverage of hyphal sample represented the percentage of each gene covered by reads. The different colors in the figure represent the proportion of genes that have a certain coverage range. C. Tags length distribution of spore sample. D. Tags length distribution of hyphal sample.

For the lncRNA-mRNA transcriptome, 1,567,771,788 high-quality clean reads (Q30 > 93%) were retained after filtering raw data (1,570,717,414 reads; Suppl. material 2: table S1). Base composition and coverage analyses indicated uniform genomic distribution without 3′/5′ bias, with > 77% of reads exhibiting ≥ 80% gene coverage (Fig. 2A, B). Sequencing saturation analysis confirmed adequate depth for capturing expressed genes (Suppl. material 1: fig. S2).

Small RNA sequencing yielded 136,978,282 clean reads (Suppl. material 2: table S1), with distinct size distributions: 22-nt RNAs dominated in spores, whereas 23-nt RNAs were predominant in hyphae (Fig. 2C, D). After removing known non-coding RNAs in GeneBank (rRNA, scRNA, snoRNA, snRNA, and tRNA), 80,548,874 unmapped tags were retained (Suppl. material 1: fig. S3A). Subsequent genome alignment identified 102,779,029 mapped tags, including 7,548,666 exon-matching tags (Suppl. material 1: fig. S3B, C), with no repeat sequence matches detected (Suppl. material 1: fig. S3D). Collectively, these results demonstrate high-quality, reproducible sequencing data suitable for downstream bioinformatic analyses.

### ﻿Analysis of mRNAs

#### ﻿Identification and characterization of mRNAs

**Figure 3.**
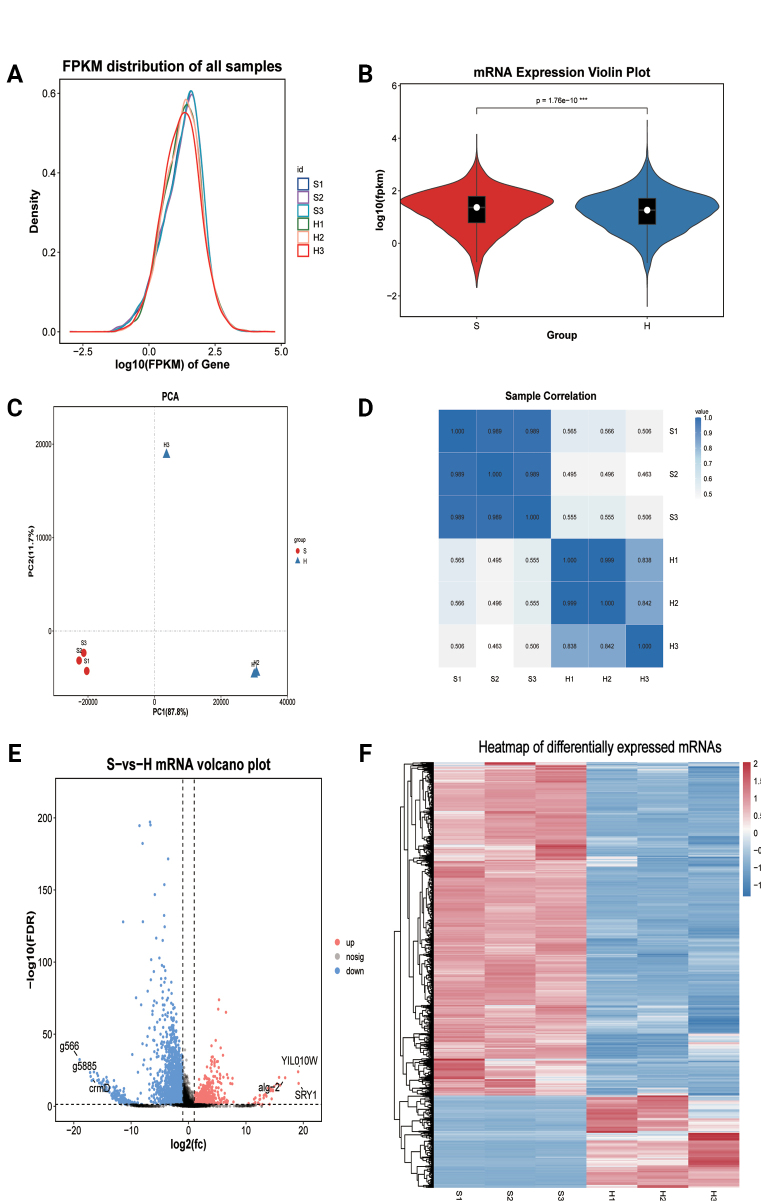
Quality assessment and differential expression analysis of the mRNA sequencing data. A. FPKM distribution. B. Gene expression violin plot. *p* = 1.76E-10***. C. Sample principal component analysis (PCA). PC 1 and PC 2 coordinates represented the first and second principal components, respectively, and the percentages in parentheses represented their respective contributions to the sample difference. D. Sample correlation heat map. The horizontal and vertical coordinates in the graph were for each sample, and the shade of the color indicated the magnitude of the correlation coefficient between the two samples (blue, high correlation. white, low correlation. E. Volcano plot of mRNAs. The horizontal coordinate denoted the log value of the multiplicative difference between the two groups, and the vertical coordinate represented the FDR negative log10 value of the difference between the two groups (red, up-regulated expression. blue, down-regulated expression). F. Differential comparison cluster heatmap. Each column represented one sample, each row represented one mRNA, and mRNAs in different samples were denoted by red (higher expression) and blue (lower expression).

To identify unannotated transcripts, we performed *de novo* transcriptome assembly using StringTie. Alignment of reconstructed transcripts to the *T. mentagrophytes* genome revealed 8,059 mRNAs, comprising 7,554 known and 505 novel transcripts (Suppl. material 2: table S2). Expression levels were quantified using FPKM (Fig. 3A), and violin plots demonstrated distinct mRNA expression patterns between spores and hyphae (Fig. 3B). Principal component analysis (PCA) and correlation analysis further confirmed clear separation between spore and hyphal groups (Fig. 3C, D).

Differential expression analysis (FDR < 0.05, |log2FC| > 1) identified 3,193 significantly differentially expressed mRNAs, including 692 up-regulated and 2,501 down-regulated transcripts in hyphae compared to spores (Suppl. material 2: table S2). Notably, the most highly up-regulated mRNAs included *SRY1* (g3940), *YIL010W* (*g3025*), and *alg2* (*g3846*), while the most strongly down-regulated transcripts included *g566*, *g5885*, and *crmD* (*g3749*) (Fig. 3E). Hierarchical clustering of differentially expressed mRNAs clearly distinguished spore and hyphal samples, with high reproducibility among biological replicates (Fig. 3F).

#### ﻿Functional enrichment analysis of mRNAs

**Figure 4.**
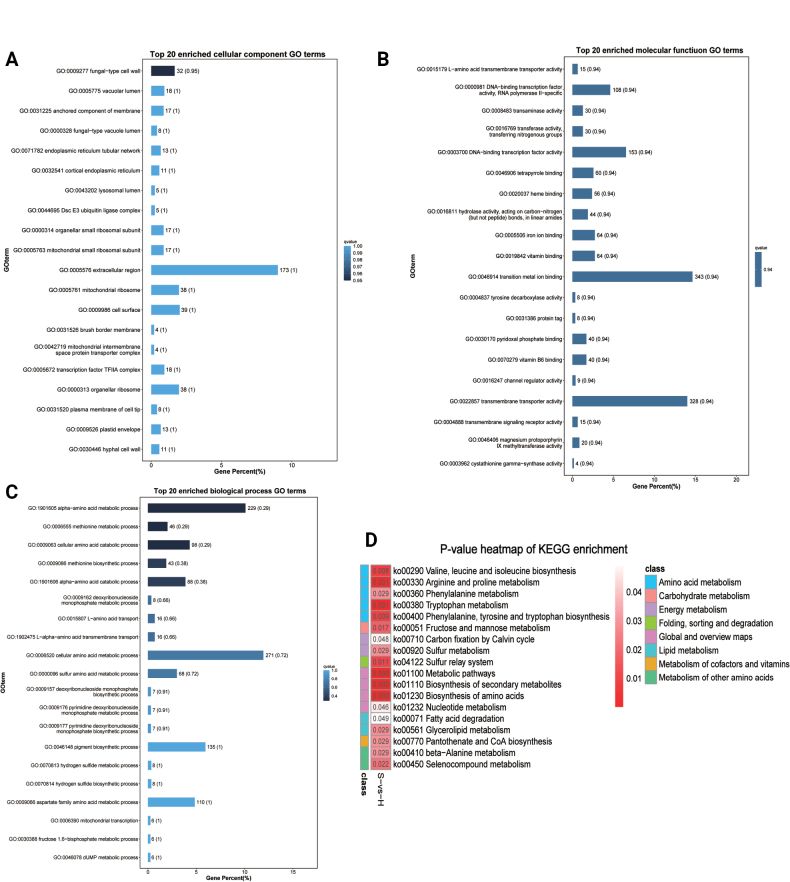
GO and KEGG classification of mRNAs. A. The top 20 enriched GO terms of the cellular component. B. The top 20 enriched GO terms of the molecular function. C. The top 20 enriched GO terms of the biological process. D. *P*-value heatmap of KEGG enrichment. S refers to the spore group and H refers to the hyphal group. The colour scale indicates *p* - values. Darker colours indicate more significant enrichment.

Functional enrichment analysis of differentially expressed mRNAs revealed significant enrichment in 292 functional categories (*P* < 0.05), comprising 31 cellular component terms (Fig. 4A; Suppl. material 2: table S3), 57 molecular function terms (Fig. 4B; Suppl. material 2: table S3), and 204 biological process terms (Fig. 4C; Suppl. material 2: table S3). Notably enriched terms included fungal-type cell wall organization, vacuolar lumen, membrane anchoring components, DNA-binding transcription factor activity (RNA polymerase II-specific), glucan exo-1,3-beta-glucosidase activity, cellular amino acid metabolism, pigment biosynthesis, mitochondrial transcription, and fructose 1,6-bisphosphate metabolism, suggesting these mRNAs play crucial roles in sporulation and biosynthetic processes.

KEGG pathway analysis further identified 18 significantly enriched metabolic pathways (*P* < 0.05; Fig. 4D; Suppl. material 2: table S3). The predominant pathways included: Amino acid metabolism, Carbohydrate metabolism, Energy metabolism, Lipid metabolism, and Metabolism of cofactors and vitamins. These findings strongly implicate the differentially expressed mRNAs in fundamental biosynthetic and metabolic processes critical for fungal development.

#### ﻿Gene set enrichment analysis of mRNAs

**Figure 5.**
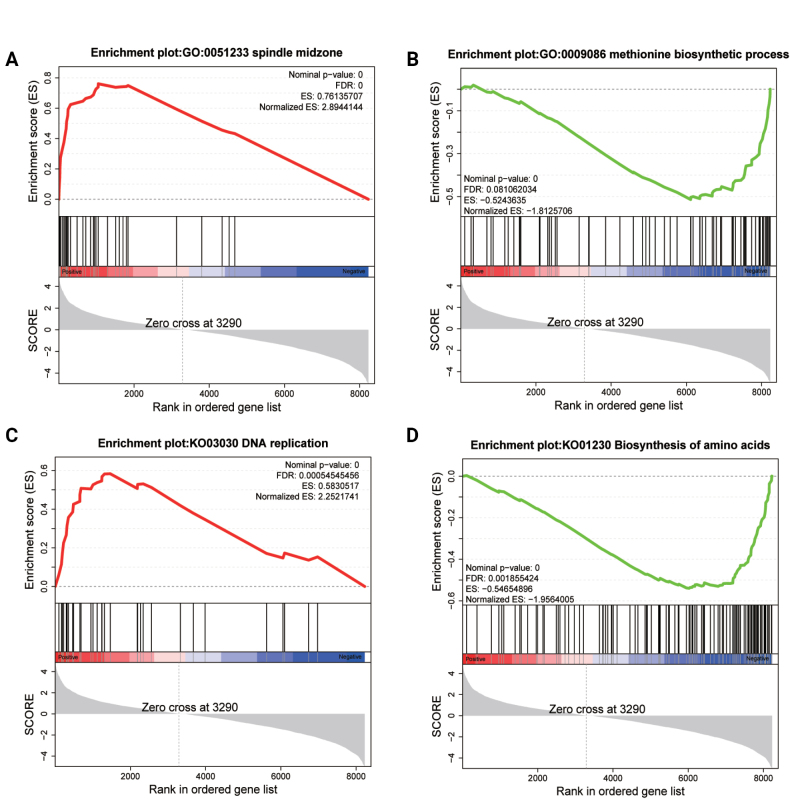
GSEA of differentially expressed mRNAs. In GO - based list, spindle midzone was enriched in hyphal groups (A) and methionine biosynthetic process was enriched in spore groups (B). In KEGG - based list, DNA replication was enriched in hyphal groups (C) and Biosynthesis of amino acids was enriched in spore groups (D).

To elucidate the functional relevance of non-significant differentially expressed mRNAs, Gene Set Enrichment Analysis (GSEA) was performed. This revealed significant enrichment of 755 GO terms (Suppl. material 2: table S4) and 23 KEGG pathways (Suppl. material 2: table S4) (|NES| > 1, NOM *p* < 0.05). Upregulated GO terms predominantly implicated mitotic processes, including spindle midzone, microtubule polymerization, mitotic spindle midzone, microtubule polymerization or depolymerization, nucleus localization, and mitotic sister chromatid segregation (Fig. 5A). Conversely, downregulated GO terms were enriched for amino acid biosynthesis (e.g., methionine biosynthetic process, cysteine biosynthetic process, sulfur amino acid biosynthetic process, proline biosynthetic process, arginine biosynthetic process, and leucine biosynthetic process) and mitochondrial ribosome assembly (e.g., mitochondrial small ribosomal subunit) (Fig. 5B). KEGG analysis further demonstrated that upregulated pathways were linked to spore germination/early outgrowth and cell division (DNA replication, Cell cycle – yeast, and Meiosis – yeast; Fig. 5C), while downregulated pathways centered on amino acid metabolism (Biosynthesis of amino acids, Arginine biosynthesis, Valine, leucine and isoleucine biosynthesis, Phenylalanine, tyrosine and tryptophan biosynthesis) and central carbon metabolism (Terpenoid backbone biosynthesis, Alanine, aspartate and glutamate metabolism, 2-Oxocarboxylic acid metabolism, and Citrate cycle (TCA cycle); Fig. 5D). Collectively, during *T. mentagrophytes* germination, upregulated genes drive cell division and cytoskeletal reorganization, whereas downregulated genes suppress anabolic processes including amino acid and terpenoid biosynthesis.

#### ﻿Protein-protein interaction network analysis of mRNAs

**Figure 6.**
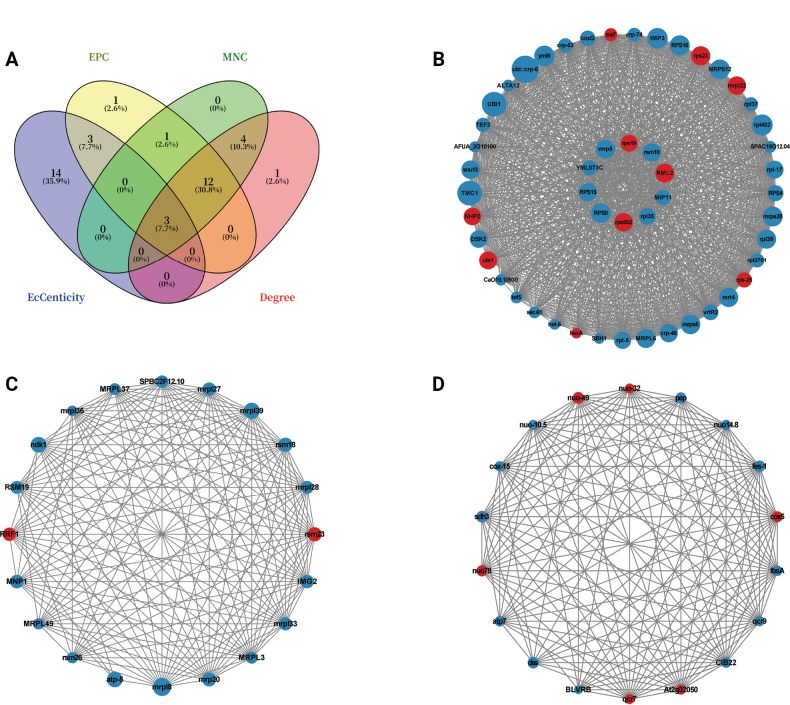
Protein-protein interaction network analysis of mRNAs. A. Identification of hub genes by four algorithms (Degree, EPC, Eccentricity, and MNC). B–D. Three representative clusters. Red indicated up-regulated genes, blue indicated down-regulated genes.

To identify functionally significant protein-protein interactions (PPI) among differentially expressed mRNAs, a PPI network was constructed using the STRING database and visualized in Cytoscape. The network comprised 1,278 nodes and 9,124 edges (Suppl. material 2: table S5). Hub genes were prioritized through intersection analysis of four topological algorithms (Degree, EPC, Eccentricity, and MNC), identifying *g2724* (*mrps28*), *g4817* (*rps23*), and *g590* (*yml6*) as the top three candidates (Fig. 6A). Subsequent MCODE clustering revealed 16 significant modules (Suppl. material 2: table S5), with the three highest-scoring clusters being Cluster 1 (51 nodes, 1,204 edges; MCODE score = 48.16), Cluster 2 (20 nodes, 160 edges; score = 16.842), and Cluster 3 (18 nodes, 141 edges; score = 16.588) (Fig. 6B–D).

### ﻿Analysis of lncRNAs

#### ﻿Identification and characterization of lncRNAs

**Figure 7.**
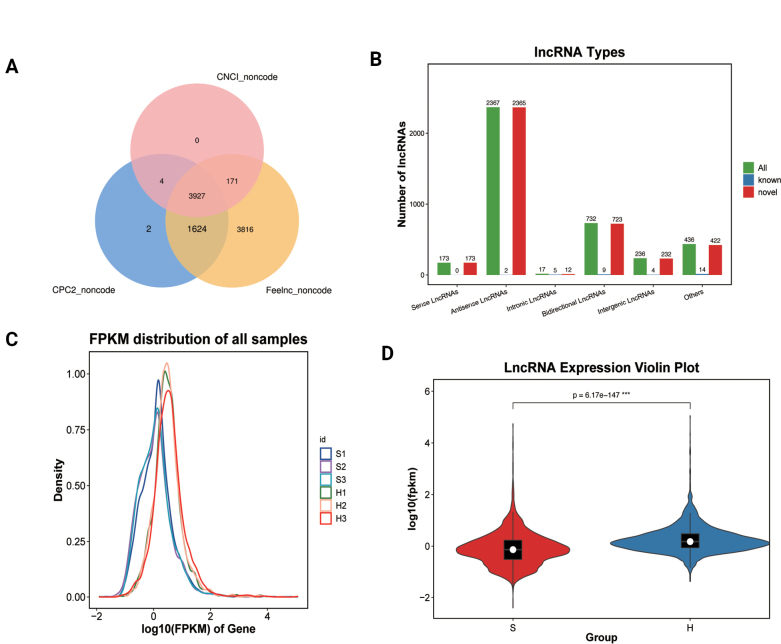
Identification and characterization of lncRNAs. A. Identification of novel lncRNAs. B. lncRNA type. C. FPKM distribution. D. lncRNA expression violin plot. *p* = 6.17E-147***.

Transcriptome assembly was performed using StringTie, followed by comprehensive protein-coding potential assessment through the intersection of FEELnc, CNCI, and CPC2 analyses. This approach identified 3,961 high-confidence lncRNAs, comprising 34 known and 3,927 novel transcripts (Fig. 7A; Suppl. material 2: table S6). Based on their genomic positions relative to protein-coding genes, the lncRNAs were categorized as: 173 sense, 2,367 antisense, 17 intronic, 732 bidirectional, 236 intergenic, and 436 other lncRNAs were identified (Fig. 7B). Transcript abundance, quantified by FPKM, revealed distinct lncRNA expression profiles (Fig. 7C). Violin plot analysis further demonstrated significantly higher lncRNA expression in hyphae compared to spores (Fig. 7D), suggesting potential regulatory roles during fungal growth and development.

#### ﻿Identification and analysis of differentially expressed lncRNAs

**Figure 8.**
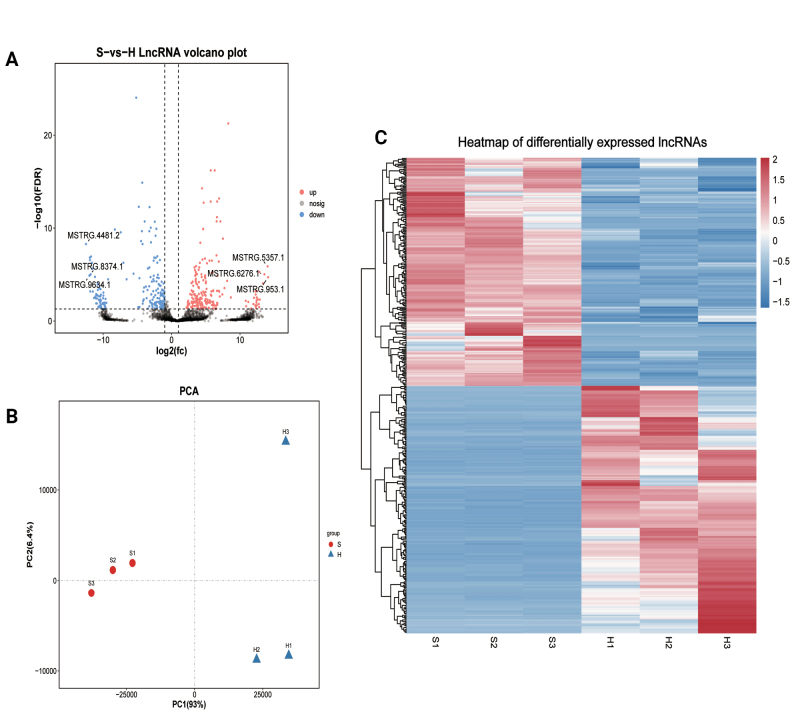
Analysis of differentially expressed lncRNAs. A. Volcano plot of lncRNAs. B. Principal component analysis. C. Heatmap of differentially expressed lncRNAs.

Comparative analysis between spore and hyphal groups identified 409 differentially expressed lncRNAs (FDR < 0.05, |log2FC| > 1), with 213 upregulated and 196 downregulated transcripts (Suppl. material 2: table S6). Notably, the most significantly upregulated lncRNAs included *MSTRG.953.1*, *MSTRG.5357.1*, and *MSTRG.6276.1*, while the most downregulated were *MSTRG.4481.2*, *MSTRG.9634.1*, and *MSTRG.8374.1* (Fig. 8A).

PCA revealed clear separation between spore and hyphal groups (Fig. 8B), confirming distinct lncRNA expression patterns. This differential expression profile was further supported by hierarchical clustering analysis, where the six samples consistently grouped according to their developmental stages (Fig. 8C). The robust segregation observed in both PCA and heatmap analyses underscores the stage-specific regulation of lncRNAs during fungal morphogenesis.

#### ﻿Functional enrichment analysis of *antisense* lncRNAs

**Figure 9.**
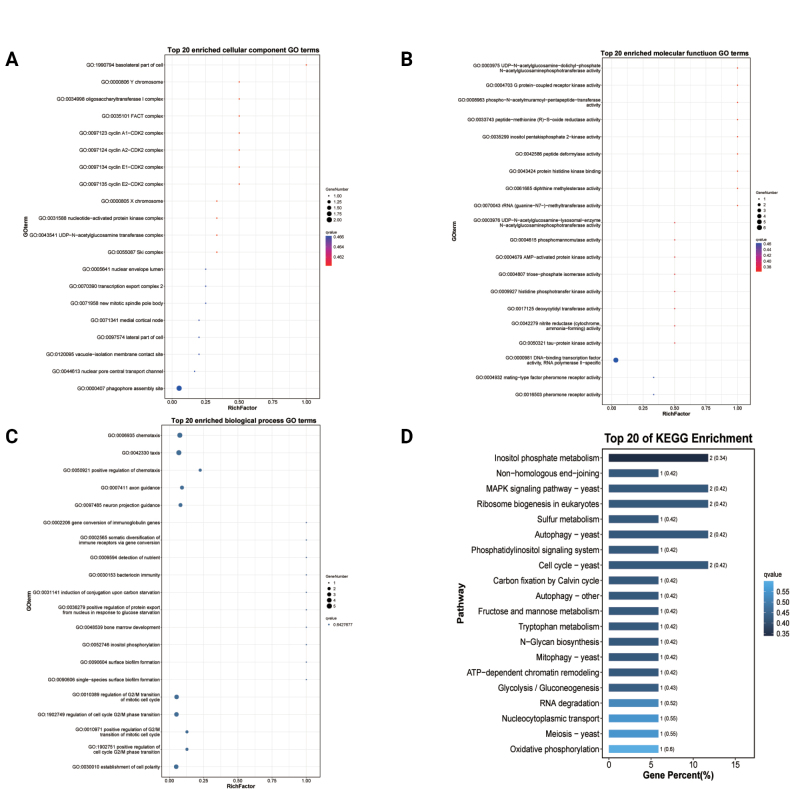
GO and KEGG classification of *antisense*lncRNAs – target mRNAs. A. Top 20 GO terms enriched by cellular components. B. Top 20 GO terms enriched by molecular function. C. Top 20 GO terms enriched by biological process. D. Top 20 pathways of KEGG enrichment analysis.

Emerging evidence suggests lncRNAs may regulate DNA demethylation, transcription, and mRNA stability through antisense mechanisms (Liu et al. 2021). Our RNAplex analysis identified 99 high-confidence *antisense* binding pairs between 97 differentially expressed *antisense*lncRNAs and their corresponding target mRNAs (Suppl. material 2: table S7). Functional annotation of these target mRNAs revealed significant enrichment in 138 GO terms (*P* < 0.05), including: 18 cellular component terms (e.g., novel mitotic spindle pole body, structural constituent of cell wall) (Fig. 9A; Suppl. material 2: table S7), 24 molecular function terms (including G protein-coupled receptor kinase activity, AMP-activated protein kinase activity, and glutaminase activity) (Fig. 9B; Suppl. material 2: table S7), and 105 biological process terms (particularly positive regulation of G2/M transition, establishment of cell polarity, and differentiation-related morphogenesis) (Fig. 9C; Suppl. material 2: table S7). These findings strongly suggest antisense lncRNAs play crucial roles in mitotic regulation and spore activation. KEGG pathway analysis further supported these observations, with enrichment in: Yeast MAPK signaling pathway, Eukaryotic ribosome biogenesis, Yeast cell cycle regulation, Oxidative phosphorylation, Amino acid biosynthesis, and Secondary metabolite biosynthesis (Fig. 9D; Suppl. material 2: table S7). The coordinated enrichment of these pathways highlights the potential involvement of antisense lncRNAs in fundamental cellular processes during fungal development and morphogenesis.

#### ﻿Functional enrichment analysis of *cis*-lncRNAs

Previous studies have established that lncRNAs can *cis*-regulate adjacent genes on the same allele (Yan et al. 2017). Applying a 10 kb genomic window (upstream or downstream) to identify potential *cis*-regulatory lncRNAs, we identified 1,158 significant *cis*-interactions involving 389 differentially expressed lncRNAs and 950 target mRNAs (Suppl. material 2: table S8).

**Figure 10.**
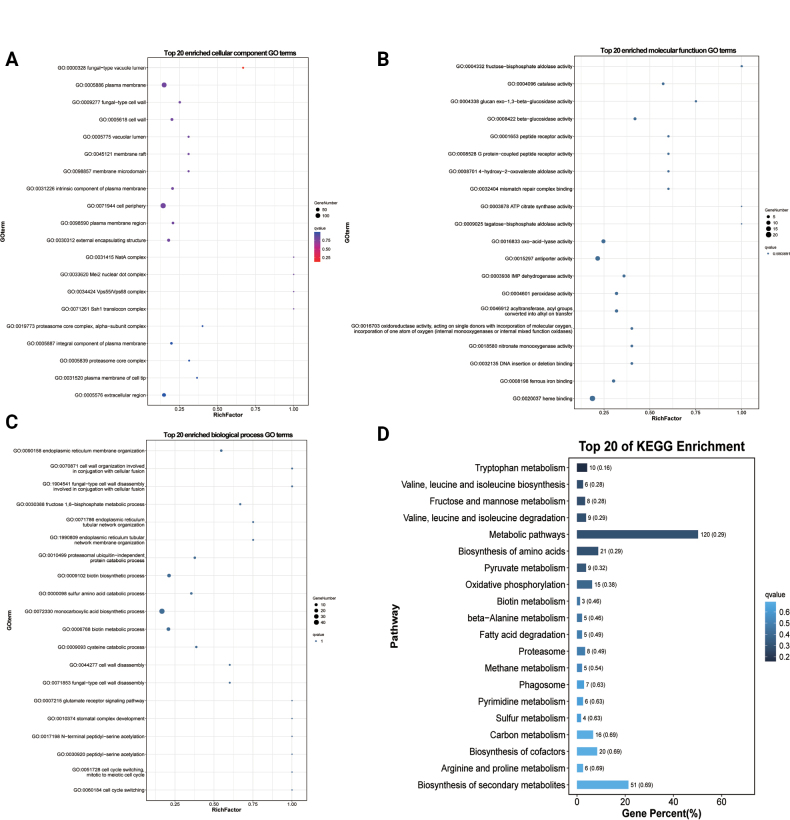
GO and KEGG classification of *cis*-lncRNAs target mRNAs. A. The top 20 enriched GO terms of the cellular component. B. The top 20 enriched GO terms of the molecular function. C. The top 20 enriched GO terms of the biological process. D. Top 20 of KEGG enrichment analysis.

Functional enrichment analysis of these *cis*-regulated target mRNAs revealed significant enrichment in 160 functional categories (*P* < 0.05), comprising: 28 cellular component terms (e.g., fungal-type vacuole lumen, cell wall components), 50 molecular function terms (including fructose-bisphosphate aldolase activity, G protein-coupled peptide receptor activity, and ATP citrate synthase activity), and 82 biological process terms (notably fungal-type cell wall disassembly during cellular fusion, fructose 1,6-bisphosphate metabolism, and cell cycle regulation) (Fig. 10A–C; Suppl. material 2: table S8). These enriched terms strongly implicate *cis*-acting lncRNAs in critical developmental processes, particularly hyphal formation and spore activation. KEGG pathway analysis further demonstrated enrichment in key metabolic pathways (*P* < 0.05), including: Amino acid metabolism (tryptophan, valine/leucine/isoleucine biosynthesis), Carbohydrate metabolism (fructose and mannose), Energy metabolism (oxidative phosphorylation) (Fig. 10D; Suppl. material 2: table S8). Collectively, these findings demonstrate that *cis*-acting lncRNAs participate in fungal development by locally regulating genes involved in cell wall remodeling, metabolic reprogramming, and cellular differentiation during hyphae formation and spore activation.

#### ﻿Functional enrichment analysis of *trans*-lncRNAs

**Figure 11.**
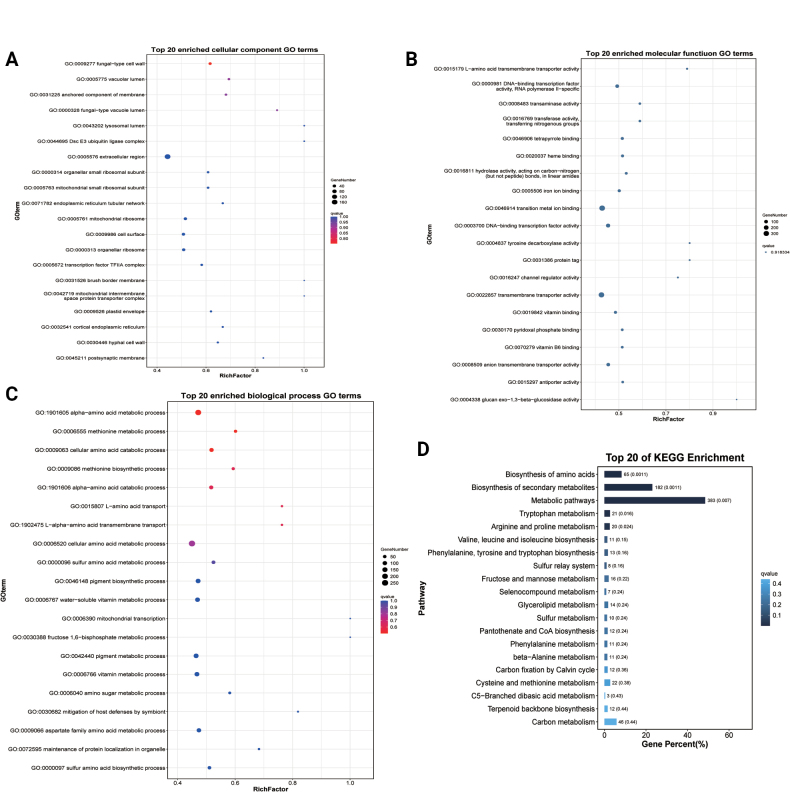
GO and KEGG classification of *trans*-lncRNAs target mRNAs. A. The top 20 enriched GO terms of the cellular component. B. The top 20 enriched GO terms of the molecular function. C. The top 20 enriched GO terms of the biological process. D. Top 20 of KEGG enrichment analysis.

In addition to *cis*-regulation, lncRNAs can modulate gene expression through *trans*-acting mechanisms (Yan et al. 2017). Our analysis identified 247,586 significant co-expression relationships between 409 differentially expressed lncRNAs and 3,138 target mRNAs (Suppl. material 2: table S9). Functional enrichment of these *trans*-regulated targets revealed significant associations (*P* < 0.05) with key biological processes, including: 30 cellular component terms (e.g., fungal-type cell wall, vacuolar lumen, hyphal cell wall), 53 molecular function terms (such as L-amino acid transmembrane transporter activity and DNA-binding transcription factor activity), and 192 biological process terms (including alpha-amino acid metabolic process, pigment biosynthetic process, and adhesion of symbiont to host) (Fig. 11A–C; Suppl. material 2: table S9). KEGG pathway analysis further identified 17 significantly enriched metabolic pathways (*P* < 0.05), notably encompassing amino acid biosynthesis (tryptophan, arginine/proline, and branched-chain amino acids), carbohydrate metabolism (fructose and mannose), lipid metabolism (glycerolipids metabolism), and cofactor synthesis (pantothenate and CoA biosynthesis) (Fig. 11D; Suppl. material 2: table S9). These comprehensive findings demonstrate that *trans*-acting lncRNAs likely orchestrate fungal development by globally regulating genes involved in cell wall organization, metabolic reprogramming, and host-microbe interactions.

### ﻿Analysis of miRNAs

#### ﻿Identification and characterization of miRNAs

Our small RNA sequencing analysis identified 733 known miRNAs (miRBase-matching sequences) through alignment with the miRBase database (Suppl. material 2: table S10) and predicted 52 novel miRNAs based on genomic positioning and hairpin structure analysis (Suppl. material 2: table S10). Both known and novel miRNAs exhibited a characteristic 5’ uracil bias in the 18–25 nt size range across spore and hyphal stages (Suppl. material 1: fig. S4A–D), with position-specific nucleotide preferences consistent with canonical miRNA features (Suppl. material 1: fig. S4E–H). Comprehensive annotation of 129,717,858 small RNA tags revealed diverse RNA species, including 44.5 million rRNAs, 7.5 million exon-mapping sequences, 574,139 known miRNAs, and 38,066 novel miRNAs (Suppl. material 1: fig. S5A). Notably, TPM-based expression analysis revealed significant differences in miRNA abundance between spores and hyphae (Suppl. material 1: fig. S5B), suggesting stage-specific regulatory roles during fungal development.

#### ﻿Identification and analysis of differentially expressed miRNAs

**Figure 12.**
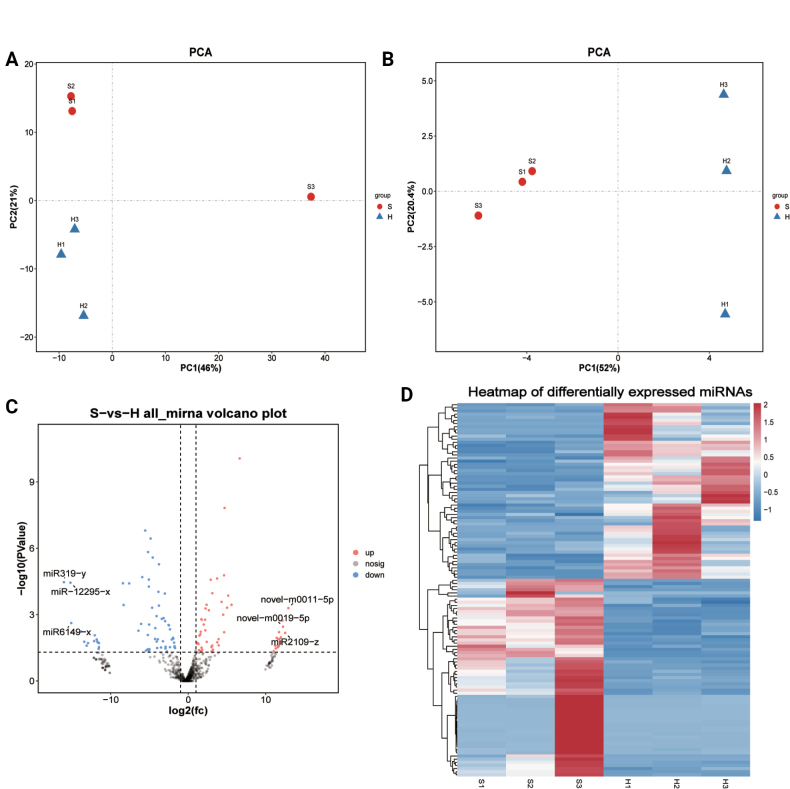
Identification and characterization of miRNAs. A. PCA of known miRNAs. B. PCA of novel miRNAs. C. Volcano plot of miRNAs. D. Heatmap of differentially expressed miRNAs.

PCA revealed distinct clustering patterns between spore and hyphal groups for both known and novel miRNAs (Fig. 12A, B). Differential expression analysis identified 119 significantly differentially expressed miRNAs (*P* < 0.05, |log2FC| > 1), comprising 56 upregulated and 63 downregulated species (Suppl. material 2: table S10). Notably, novel-m0011-5p, miR2109-z, and novel-m0019-5p emerged as the most strongly upregulated miRNAs, while miR319-y, miR-12295-x, and miR6149-x showed the most pronounced downregulation (Fig. 12C). Hierarchical clustering of these differentially expressed miRNAs clearly distinguished hyphal from spore samples (Fig. 12D), demonstrating robust stage-specific miRNA expression profiles during fungal development.

#### ﻿Functional enrichment analysis of miRNAs

**Figure 13.**
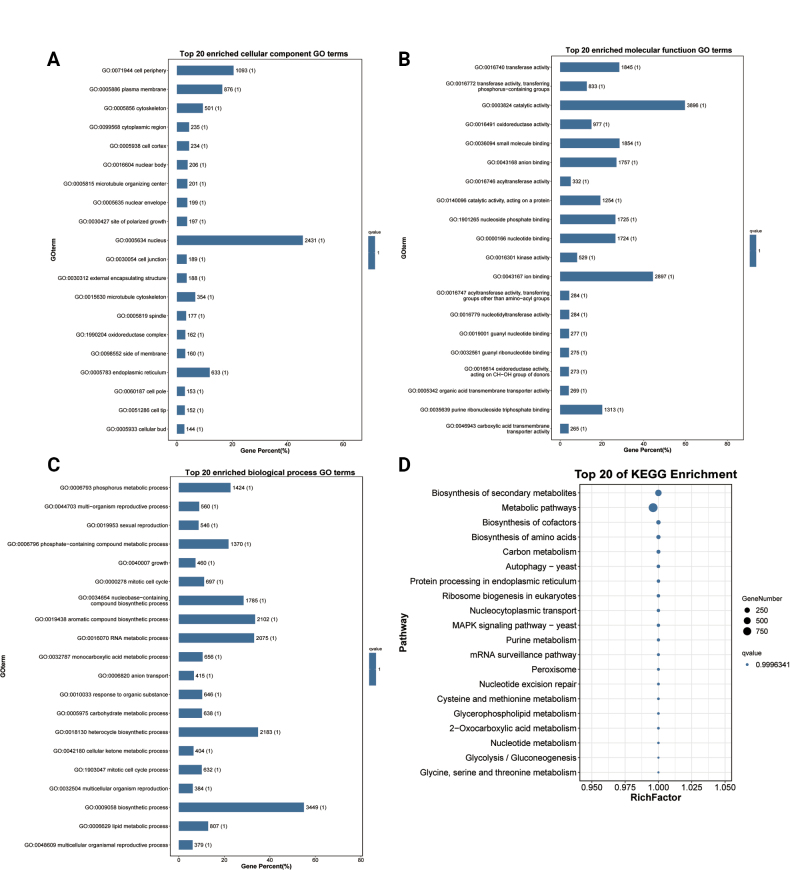
GO and KEGG classification of miRNAs target mRNAs. A. Top 20 GO terms enriched by cellular components. B. Top 20 GO terms enriched by molecular function. C. Top 20 GO terms enriched by biological process. D. Top 20 pathways of KEGG enrichment analysis.

Through comprehensive target prediction, we identified 8,662 mRNAs potentially regulated by 785 miRNAs, encompassing 626,128 target sites (Suppl. material 2: table S11). Functional enrichment analysis of these miRNA-targeted mRNAs revealed significant associations (*P* < 0.05) with 19 functional categories, including plasma membrane (cellular component), transferase/oxidoreductase activity (molecular function), and key biological processes such as multi-organism reproduction, growth, and mitotic cell cycle regulation (Fig. 13A–C; Suppl. material 2: table S11). KEGG pathway analysis further demonstrated enrichment in 140 metabolic and signaling pathways, most notably biosynthesis-related processes, including secondary metabolites, amino acids, and cofactors, as well as ribosome biogenesis and yeast MAPK signaling (Fig. 13D; Suppl. material 2: table S11). These findings strongly suggest that miRNAs play a pivotal role in coordinating fungal growth, development, and metabolic adaptation through widespread post-transcriptional regulation.

### ﻿Analysis of ceRNAs

#### ﻿Construction of a candidate ceRNA network

**Figure 14.**
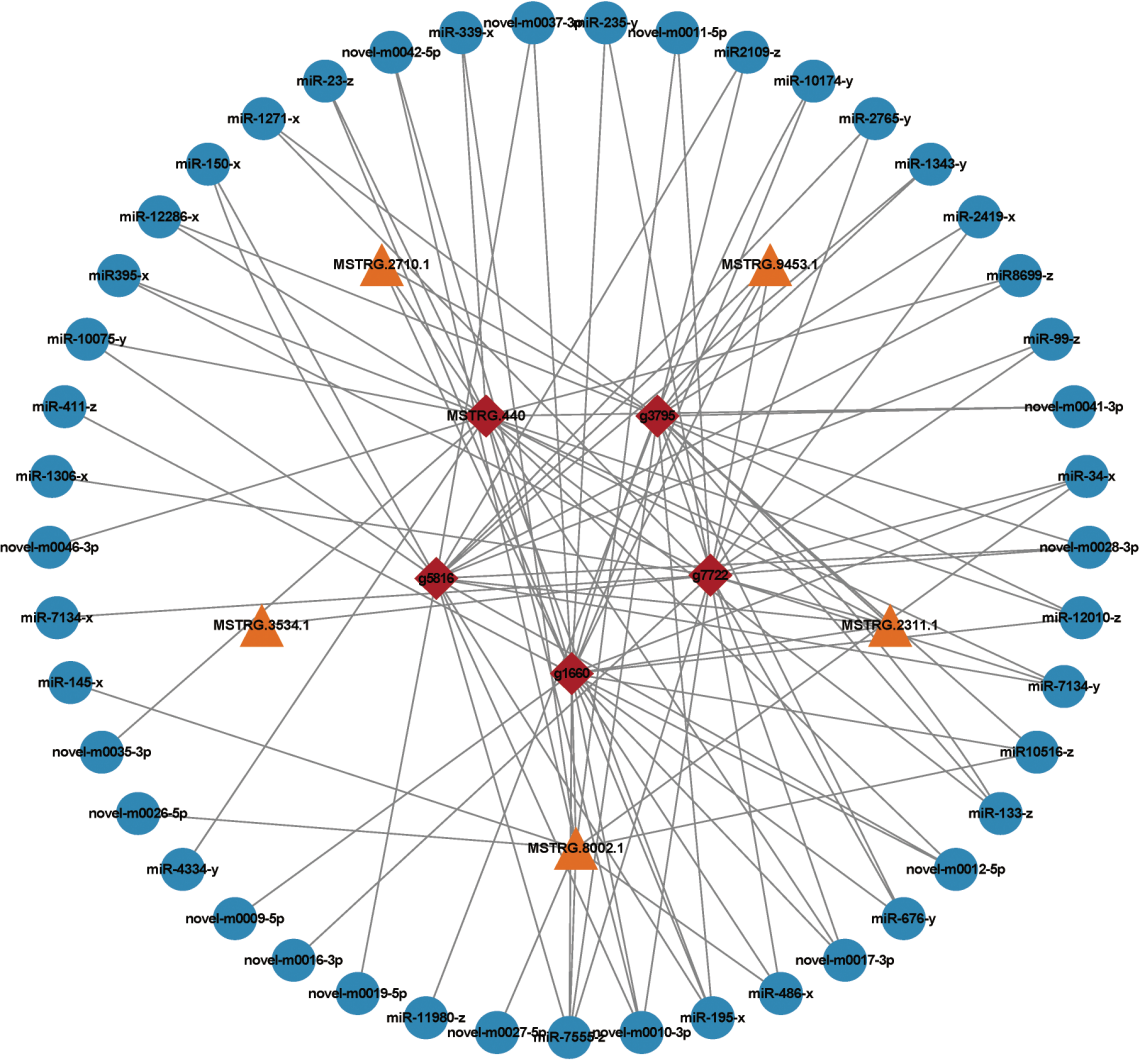
The highest connectivity for 5 mRNAs and 5 lncRNAs. Red represented mRNAs, yellow represented lncRNAs, and blue represented miRNAs.

Through comprehensive target prediction, we identified 34,680 miRNA-mRNA and 3,227 miRNA-lncRNA interaction pairs involving 119 differentially expressed miRNAs, 3,177 mRNAs, and 403 lncRNAs. Using stringent correlation thresholds (*SCC* ≤ -0.7 for negative co-expression and *PCC* > 0.9 for ceRNA pairs), we derived 229,789 potential ceRNA interactions between 2,911 mRNAs and 338 lncRNAs. For statistical screening of high-confidence pairs, we performed hypergeometric testing and retained interactions with an uncorrected *P*-value < 0.05 (note: multiple-testing correction such as FDR adjustment was not applied in this step), ultimately yielding a candidate network comprising 14,360 significant ceRNA pairs, integrating 2,627 mRNAs, 107 miRNAs, and 329 lncRNAs (Suppl. material 2: table S12). Topological analysis revealed 636 hub nodes in the primary network tier, with the highest connectivity observed for five mRNAs (*glt1*, *dtxS1*, *g3795*, *SPBC20F10.07*, and *MSTRG.440*) and five lncRNAs (*MSTRG.9453.1*, *MSTRG.2311.1*, *MSTRG.2710.1*, *MSTRG.3534.1* and *MSTRG.8002.1*), along with their miRNA partners (Fig. 14), highlighting central regulators in the fungal ceRNA network.

#### ﻿Exploratory enrichment analysis of ceRNAs

**Figure 15.**
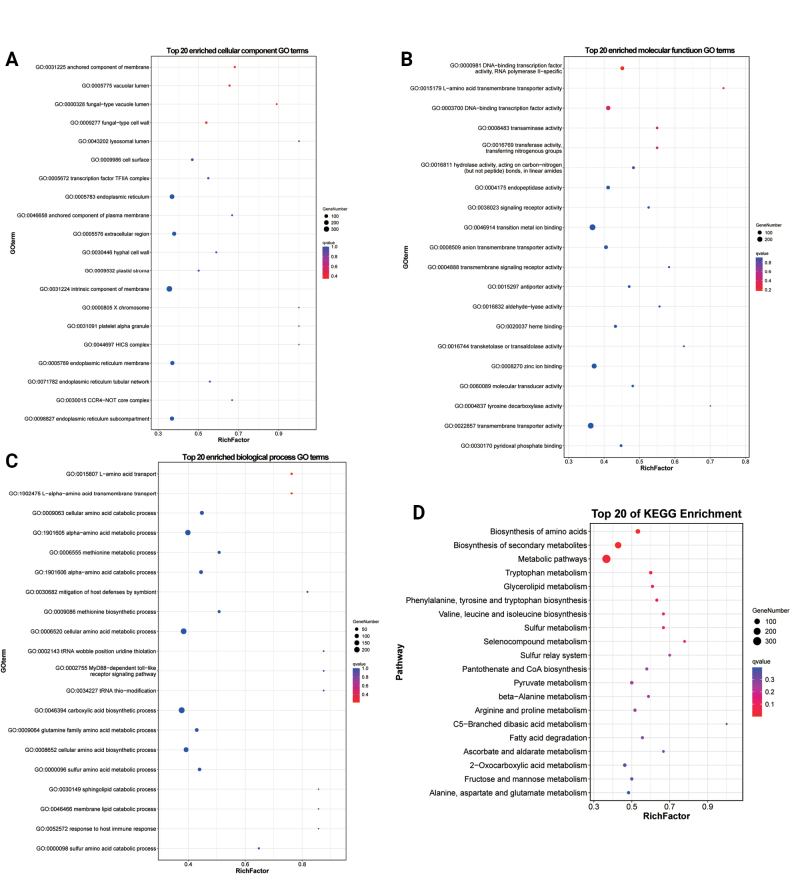
GO and KEGG classification of ceRNA-related mRNAs. A. Top 20 GO terms enriched by cellular components. B. Top 20 GO terms enriched by molecular function. C. Top 20 GO terms enriched by biological process. D. Top 20 pathways of KEGG enrichment analysis.

Functional annotation of ceRNA-related mRNAs revealed significant enrichment (uncorrected *P*-values < 0.05) in 286 categories (32 cellular components, 68 molecular functions, and 186 biological processes), including fungal-type vacuole lumen, fungal-type cell wall, hyphal cell wall, L-amino acid transmembrane transporter activity, DNA-binding transcription factor activity, L-amino acid transport, methionine metabolic process, and cellular amino acid biosynthetic process (Fig. 15A–C; Suppl. material 2: table S12). KEGG pathway analysis further identified 18 significantly enriched metabolic pathways (uncorrected *P*-values < 0.05), with predominant representation in amino acid biosynthesis (tryptophan), secondary metabolite production, and central carbon metabolism (glycerolipid and pyruvate metabolism) (Fig. 15D; Suppl. material 2: table S12). These findings provide preliminary clues that lncRNAs modulate fungal development and metabolism through miRNA-mediated ceRNA networks, competitively regulating mRNAs involved in cell wall dynamics and metabolic reprogramming.

### ﻿qRT-PCR verification of the RNA-Seq results

**Figure 16.**
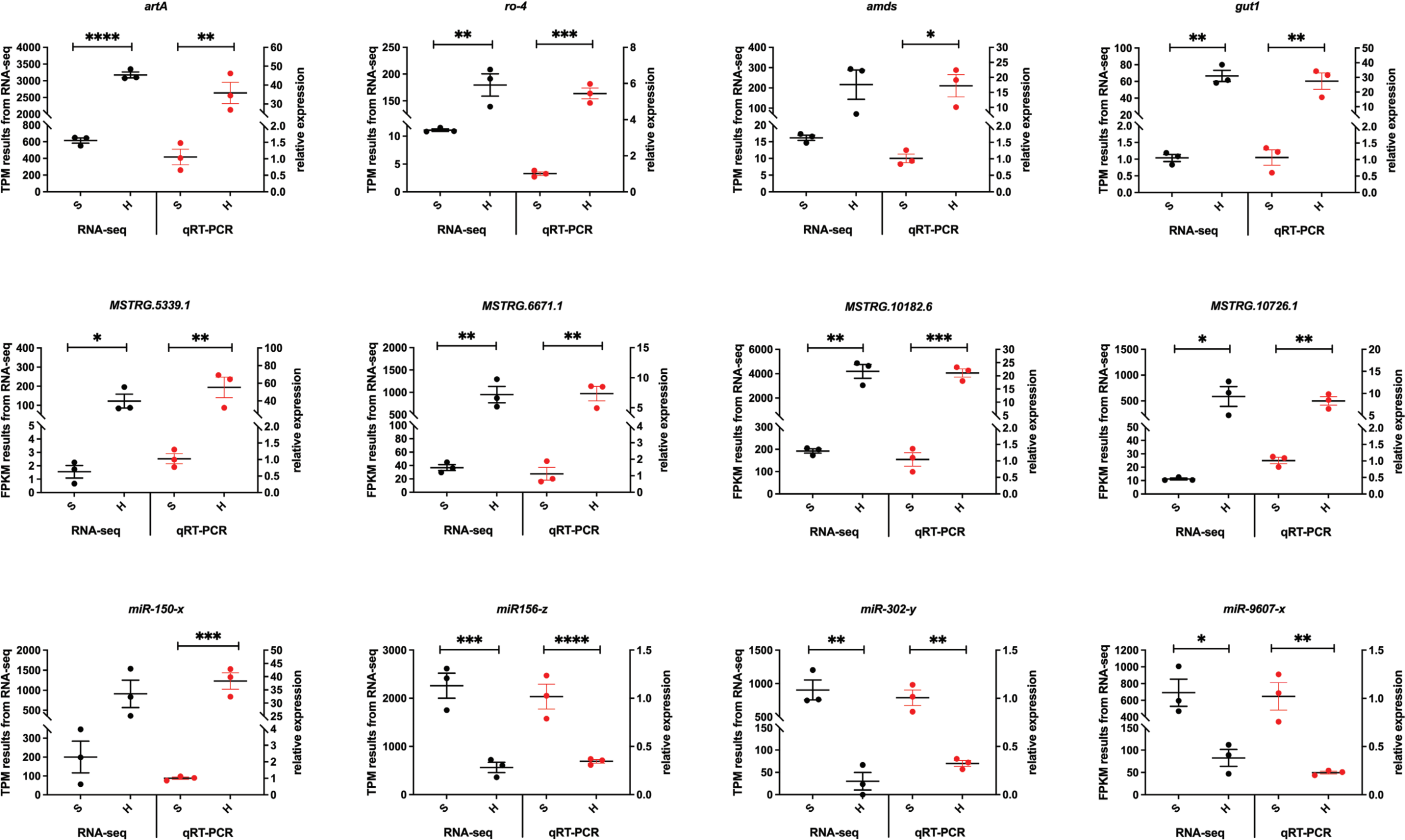
qRT-PCR verification of the RNA-Seq Results. Upper panel, mRNAs. Middle panel, lncRNAs. Lower panel, miRNAs. Data are shown as mean ± SEM. *p < .05, **p < .01, ***p < .001 and ****p < .0001.

To validate the RNA sequencing data, qRT-PCR was performed on a randomly selected panel of differentially expressed mRNAs, lncRNAs, and miRNAs using primers designed via Primer-BLAST (Suppl. material 2: table S13). Expression trends of mRNAs and lncRNAs exhibited significant concordance with RNA-seq profiles (Fig. 16; Suppl. material 2: table S13). Among miRNAs, miR-150-x showed significant upregulation during germination, whereas three others were down-regulated (Fig. 16). Critically, the positive correlation between up-regulated lncRNA-*MSTRG.10182.6* and up-regulated target gene *ro-4* aligned with the predicted ceRNA network interaction. These results collectively confirm the reliability of our transcriptomic data and the accuracy of identified expression dynamics.

## ﻿Discussion

The transition from dormant spores to invasive hyphae in dermatophytes (e.g., *Trichophyton rubrum*, *T. mentagrophytes*) is orchestrated by multilayered molecular controls, including transcriptional, post-transcriptional, and epigenetic regulation. This process involves dynamic interactions between mRNAs, miRNAs, and lncRNAs, which collectively fine-tune fungal development and pathogenicity (Liu et al. 2007; Wang et al. 2018; Zhang et al. 2023). In the present study, we present a comprehensive transcriptomic landscape of the spore-to-hypha transition in *T. mentagrophytes* by deploying multi-stage RNA-seq. We systematically profiled the dynamic expression of stage-specific mRNAs, lncRNAs, and miRNAs throughout this critical morphogenetic process. Furthermore, we constructed putative competing endogenous RNA (ceRNA) networks to elucidate the post-transcriptional crosstalk mediated by lncRNAs.

This study represents a significant expansion beyond our earlier circRNA-focused investigation (Zhang et al. 2023). While that work laid the groundwork by identifying circRNAs as potential regulatory elements, the present multi-transcriptome analysis substantially broadens the regulatory landscape by integrating mRNA, lncRNA, and miRNA expression dynamics. This more holistic perspective has been crucial, as it enabled us to move beyond the role of individual RNA species and uncover their complex interactions within putative ceRNA networks. Consequently, our current work provides a first systems-level view of the transcriptional circuitry governing dermatophyte morphogenesis.

### ﻿Germination dynamics and transcriptional reprogramming

The germination of *T. mentagrophytes* spores progresses through distinct morphological phases: initial spore swelling (0–6 h), germ tube emergence (12 h), and hyphal maturation (18 h), consistent with previous reports in *Aspergillus nidulans* and *Candida* species (Osherov and May 2000; Rampitsch et al. 2012). During the spore swelling phase, dormant spores undergo rapid water uptake and expansion, doubling their cell diameter (approximately 2–3 μm in diameter), while establishing cellular polarity to prepare for subsequent polarized growth. This stage is accompanied by rapid activation of energy metabolism and cell wall reorganization. Our transcriptomic data reveal that spore swelling is associated with significant upregulation of genes related to glycolysis and oxidative phosphorylation (e.g., *pfkA* and *cox5*) (Cumsky et al. 1983; Li et al. 2021), providing the necessary ATP supply for germination. Concurrently, changes in the expression of chitin synthase (e.g., *CHS2* and *chsG*) and β-glucanase genes (e.g., *ARB_04467*) indicate dynamic cell wall remodeling to accommodate volumetric expansion (Mellado et al. 2003; Wu et al. 2024).

Upon entering the germ tube formation phase, the spore breaches its cell wall at a specific site to form a single germ tube, which ultimately develops into a mature hyphal network. This process heavily relies on cytoskeletal rearrangement and vesicular trafficking systems. Our study found that genes significantly upregulated during germ tube formation primarily involve microtubule polymerization (e.g., *tubB*), actin cytoskeleton regulation (e.g., *myo2*), and vesicle transport (e.g., *sec11* and *sec27*) (Govindan et al. 1995; Oakley 2004; Karnik et al. 2015; Friend et al. 2018). The gene expression patterns observed during this morphological transition share similarities with yeast filamentation, yet *T. mentagrophytes* exhibits stronger expression of environmental adaptation and virulence-related genes.

The observed doubling in spore diameter prior to germ tube emergence suggests substantial metabolic activation, likely involving cell wall remodeling and nutrient uptake, as seen in *Neurospora crassa* (Bowman et al. 2006; Maddi et al. 2009). RNA-seq analysis revealed 3,193 differentially expressed mRNAs (692 upregulated, 2,501 downregulated), with functional enrichment highlighting fungal-type cell wall organization, amino acid metabolism, and mitochondrial transcription. These findings align with studies in *Aspergillus fumigatus*, where germination involves rapid activation of cell wall synthases and metabolic enzymes (Bleichrodt et al. 2020). The strong downregulation of amino acid biosynthetic pathways (e.g., methionine, cysteine, and leucine biosynthesis) suggests a shift from anabolic processes to catabolic utilization of stored nutrients, a strategy also observed in *Magnaporthe oryzae* (Fernandez et al. 2014; Fernandez and Wilson 2014). Conversely, upregulated genes were enriched in mitotic processes (e.g., spindle midzone assembly, microtubule polymerization), consistent with the transition from dormancy to active growth (Harris 2005).

### ﻿LncRNAs orchestrate developmental transitions through multilayered regulation

#### ﻿*Cis*-regulatory mechanisms and their functions

We identified 389 differentially expressed lncRNAs in *T. mentagrophytes* that may regulate neighboring genes in *cis* (within a 10 kb window). These lncRNAs formed 1,158 significant regulatory pairs with 950 target mRNAs. Functional enrichment analysis revealed that *cis*-regulated target genes were significantly associated with pathways such as: cell wall remodeling (e.g., *exgD*), fructose metabolism (e.g., fructose-1,6-bisphosphate aldolase *FBA1*), and cell cycle regulation (e.g., G2/M phase regulators *CDC2* and *cdcA*) (Cieśla et al. 2014; Liu et al. 2015). For example, *MSTRG.7978.1*, located upstream of the cell wall remodeling related gene-*exgD*, was highly upregulated during hyphal growth, and its expression strongly correlated with *exgD*, suggesting it may activate *exgD* via chromatin remodeling to regulate cell wall synthesis at hyphal tips.

*Cis*-acting lncRNAs may function through multiple mechanisms: some directly bind to promoter regions of nearby genes by forming RNA-DNA triplex structures, and others act as protein scaffolds, recruiting transcription factors or chromatin-modifying complexes (Nahkuri and Paro 2012; Li et al. 2016). For instance, *MSTRG.2464.1*, adjacent to the fructose-1,6-bisphosphate aldolase *FBA1*, may enhance histone H3K27 acetylation at the target promoter by interacting with a histone acetyltransferase complex, thereby activating sugar metabolism-related genes (Cieśla et al. 2014). This mechanism resembles lncRNA *SABC1* in plants, which recruits the PRC2 complex to regulate the neighboring gene *NAC3* (Liu et al. 2022b). In *Fusarium graminearum*, lncRNA RNA5P also *cis*-regulates the trichothecene toxin gene *TRI5* via Tri6 transcription factor binding (Huang et al. 2024).

#### ﻿*Trans*-regulatory networks and their functions

Through genome-wide co-expression analysis, we constructed a *trans*-regulatory network comprising 247,586 interactions, involving 409 differentially expressed lncRNAs and 3,138 target mRNAs. These *trans*-acting lncRNAs primarily regulate pathways related to Amino acid transmembrane transport (e.g., gamma-aminobutyric acid and delta-aminolaevulinic acid permease gene *UGA4*), Pigment synthesis (e.g., cellular division, branching, and conidiation gene *aspB*), and adhesion of symbiont to host (e.g., cell-cell adhesion gene *stuA*) (Westfall and Momany 2002; Luzzani et al. 2007; Bitencourt et al. 2021). This global regulation resembles *Blakeslea trispora*, where the pheromone trisporic acid *trans*-activates terpenoid genes (*ipi*, *carG*, *carRA*, and *carB*) to boost β-carotene production during mating (Sun et al. 2012). Notably, multiple *trans*-acting lncRNAs were found to target key enzyme genes in the branched-chain amino acid biosynthesis pathway (Valine, leucine and isoleucine biosynthesis), with most genes showing downregulated expression (e.g., *LEUA*, *ILV1*, *ILV6*, *ILV2*, *ilv-2*, *ilvC*, *leu2A*, *LEU4*, and *SDL1*) (Orasch et al. 2019; Steyer et al. 2021; Steyer and Todd 2023). This phenomenon may reflect an adaptive metabolic shift in which hyphae preferentially uptake branched-chain amino acid directly via amino acid transporters in nitrogen-rich environments (e.g., liquid culture media), rather than relying on de novo synthesis.

The mechanisms of *trans*-regulation by lncRNAs are more complex: Some lncRNAs directly bind to target mRNAs via base-pairing, influencing their stability or translation efficiency. Others function as signaling hubs, integrating multiple regulatory pathways (Yoon et al. 2013; Chen and Kim 2024). For example, *MSTRG.12869.1*, which is highly expressed during hyphal growth, leads to downregulation of genes involved in L-amino acid transport (e.g., *UGA4*, *ACG1*, *MUP1*, *uba2*, *fnx2*, *ilvC*, *leu2A*, *LEU4*, and *rhb1*) (Chardwiriyapreecha et al. 2008; Kim et al. 2017; Karuppiah et al. 2023), suggesting a central role in nutrient acquisition. This global regulatory mechanism resembles *lncLSTR* in the liver, which modulates whole-body lipid metabolism via a TDP-43-mediated transcriptional network (Li et al. 2015).

### ﻿miRNA-guided post-transcriptional control

Our discovery of 119 differentially expressed miRNAs species reveals an unappreciated layer of post-transcriptional regulation in *T. mentagrophytes*. The upregulation of miR-339-x in hyphae, along with their predicted targeting of cell cycle inhibitors (e.g., *swi10*), suggests miRNAs facilitate germ tube emergence by releasing cell cycle brakes (Rödel et al. 1997). Conversely, hyphal-downregulated miRNAs (e.g., miR 159-y) may permit expression of adhesion factors (e.g., *stuA*) critical for host colonization, akin to *Histoplasma capsulatum* (Saçar Demirci 2020; Bitencourt et al. 2021). The enrichment of miRNA targets in oxidoreductase activity and secondary metabolism further indicates roles in stress adaptation during host invasion.

### ﻿Integrated ceRNA networks coordinate development

The ceRNA mechanism represents one of the pivotal regulatory modes of lncRNAs (Salmena et al. 2011). We constructed a candidate ceRNA network comprising 14,360 interaction pairs, involving 329 lncRNAs, 107 miRNAs, and 2,627 mRNAs. The network’s core hubs included 5 mRNAs (e.g., *glt1*, *dtxS1*) and 5 lncRNAs (e.g., *MSTRG.9453.1*, *MSTRG.2311.1*), all exhibiting exceptionally high connectivity (degree > 100). Functional enrichment analysis revealed that the candidate ceRNA networks were significantly associated with: fungal-type cell wall, L-amino acid transport, and Amino acid biosynthesis (e.g., methionine metabolism).

To validate the biological function of the ceRNA networks, we performed verification on the key putative ceRNA network (*MSTRG.10182.6*/miR-1388-y/*ro-4*) using qRT-PCR. The results demonstrated concurrent upregulation of both *MSTRG.10182.6* and its target gene *ro-4* (related to fungal growth) in hyphae, supporting the model wherein *MSTRG.10182.6* sequesters miR-1388-y to relieve suppression of *ro-4*, thereby promoting fungal growth (Hirozumi et al. 1999). This regulatory pattern, reminiscent of linc-MD1's action, sequesters miR-133 and miR-135 to modulate the expression of transcription factors *MAML1* and *MEF2C*, which activate muscle-specific genes (Cesana et al. 2011).

In the candidate ceRNA network, we identified a critical regulatory axis: *MSTRG.5264.1*/miR-302-y/*CHS2*, which plays a key role during the transition from spores to hyphae. In the spore stage, miR-302-y is highly expressed and suppresses the expression of *CHS2*, a gene involved in cell wall synthesis. In the hyphal stage, *MSTRG.5264.1* is upregulated and acts as a “molecular sponge” to sequester miR-302-y, thereby relieving its repression on *CHS2* and promoting cell wall synthesis (Wu et al. 2024). This regulatory mechanism resembles that of *Fusobacterium nucleatum* in colorectal cancer, where the upregulated lncRNA *EVADR* facilitates tumor metastasis (Lu et al. 2022). The similarity suggests that microbes may employ analogous RNA regulatory networks to adapt to different environments.

The putative ceRNA network is closely linked to epigenetic regulation. Our study revealed multiple putative ceRNA regulatory axes involved in modulating the expression of epigenetic-modifying enzymes (e.g., *jhd1*, *DIM1* and *jmj22*) (Fang et al. 2007). For example, the *MSTRG.12472.1*/miR-7555-z/*jhd1* axis regulates the expression of the histone demethylase gene *jhd1*. In the spore stage, high expression of *jhd1* may remove specific histone modification marks (targeting H3K36me2), regulating the transcription of developmental genes. In the hyphal stage, the lncRNA *MSTRG.11900.2* is downregulated, releasing its sequestration of miR-6149-x, thereby inhibiting *jhd1* expression (Fang et al. 2007). This alters chromatin states and regulates genes essential for hyphal growth. This crosstalk between RNA-based regulation and epigenetic modulation parallels the lncRNA SABC1/PRC2/NAC3 pathway discovered in plant immunity (Liu et al. 2022b), suggesting that conserved regulatory logic operates across diverse biological systems.

### ﻿Biological significance of key pathways and molecular targets

#### ﻿Dynamic reprogramming of amino acid metabolism

Amino acid metabolism undergoes significant dynamic reprogramming during spore germination. Our data reveal that amino acid biosynthesis-related genes are markedly downregulated in the hyphal stage, including key enzymes in the tryptophan (e.g., *trpC*, *trp*2 and *trp4*), cysteine (e.g., *cys3*, *cysD* and *cysK*), and phenylalanine (e.g., *ARO4*, *PHA2* and *aro2*) synthesis pathways (Horng et al. 1989; Sousa et al. 2002; Hiraishi et al. 2008). This downregulation likely reflects two biological imperatives: (1) the rapid growth of hyphal tips requires carbon skeleton redistribution, shifting resources from amino acid synthesis to energy production; and (2) direct utilization of environmental amino acids is more energy-efficient than de novo synthesis, a strategy analogous to the metabolic adaptation observed in pathogenic fungi during host infection.

Notably, while amino acid biosynthesis genes are generally downregulated, certain critical nodes are subject to specific lncRNA-mediated regulation. For instance, the methionine synthase gene (*MET6*) is positively regulated by *MSTRG.5357.1* within the ceRNA network, ensuring adequate supply of methyl donors to support both epigenetic modifications and protein translation during hyphal growth (Pascon et al. 2004; Chen et al. 2016). This precise regulatory mechanism parallels the lncLSTR-mediated modulation of systemic lipid homeostasis through the TDP-43/Cyp8b1/FXR/apoC2 cascade in the liver, demonstrating the conserved precision of lncRNA in metabolic regulation across biological systems (Li et al. 2015).

#### ﻿Coordination of cell cycle and morphogenesis

Spore germination requires precise coordination between cell cycle progression and morphogenesis. Our study revealed that cell cycle regulatory genes (e.g., *CDC2*, *CDCA*, *wee1*) are significantly upregulated during germ tube formation, co-expressed with genes involved in mitotic spindle assembly (e.g., *AUR1*) and sister chromatid separation (e.g., *Bub1*) (Hashida-Okado et al. 1996; Elowe 2011; Masuda et al. 2011; Liu et al. 2015). This synergy may be mediated by lncRNA *cis*-regulation. For instance, *MSTRG.8251.1*, located upstream of the cyclin gene *CDC2*, exhibits expression highly correlated with *CDC2* (both upregulated in the hyphal stage). This suggests that *MSTRG.8251.1* may facilitate promoter-enhancer interactions via chromatin looping, thereby promoting *CDC2* expression and regulating cell cycle progression (Liu et al. 2015).

Cytoskeletal reorganization is a critical process in morphogenesis. Multiple genes participate in this process, including: Microtubule-associated proteins: (*Tea2*, *StuA*, *Alp4*, *ro-4*) and Vesicle SNARE proteins (*Sec11*, *Sec27*) (Hirozumi et al. 1999; Browning and Hackney 2005; Masuda et al. 2006; Bitencourt et al. 2021). These genes are upregulated in hyphae and regulated by *trans*-acting lncRNAs, forming a complex post-transcriptional network. For example, *MSTRG.10634.1* may enhance the stability of its target mRNA (*Alp4*) by interacting with heterogeneous nuclear ribonucleoproteins, ensuring sufficient microtubule protein expression during critical germ tube formation stages (Masuda et al. 2006). This RNA-binding protein-mediated regulatory mechanism resembles the MIR100HG/hnRNPA2B1/TCF7L2 axis in colorectal cancer, highlighting evolutionarily conserved regulatory logic across diverse biological processes (Liu et al. 2022a).

### ﻿Limitations and future perspectives

This study has several technical limitations. First, only two time points were examined, leaving the dynamic changes during intermediate stages uncharacterized. Future studies should incorporate critical transitional phases (e.g., 6 h and 12 h) to elucidate the heterogeneity and developmental trajectory of spore germination. Second, the functional mechanisms of lncRNAs remain unclear. For instance, how do *cis*-acting lncRNAs precisely recognize the promoters of neighboring genes? How do *trans*-acting lncRNAs achieve target specificity? These questions warrant further investigation using protein interactome analyses (e.g., ChIRP-MS to identify binding partners). Third, although stringent criteria (SCC ≤ -0.7, PCC > 0.9) were applied to minimize false positives in ceRNA prediction, multiple-testing correction (e.g., FDR) and experimental validation of RNA-RNA interactions is essential. For example, dual-luciferase reporter assays could verify lncRNA-miRNA-mRNA targeting relationships. Finally, from a translational perspective, the identified key lncRNAs and hub genes may serve as novel targets for antifungal drug development. For instance, antisense oligonucleotides (ASOs) targeting *MSTRG.9453.1* or small-molecule inhibitors disrupting lncRNA-protein interactions could selectively inhibit spore germination without affecting host cells.

In summary, this study systematically deciphered the lncRNA-mRNA-miRNA regulatory network during *T. mentagrophytes* germination by integrating multi-omics data, revealing how lncRNAs coordinate developmental transitions through *cis*-, *trans*-, and ceRNA mechanisms. These findings provide novel insights into the regulatory framework of filamentous fungal morphogenesis and lay a theoretical foundation for RNA interference-based antifungal strategies.

## ﻿Conclusion

This study establishes a comprehensive regulatory atlas of *T. mentagrophytes* germination, revealing that: (1) Transcriptional reprogramming suppresses anabolism while activating cell division machinery. (2) lncRNAs operate through spatially distinct mechanisms (*antisense*, *cis*, and *trans*) to control morphogenesis. (3) miRNAs fine-tune developmental transitions by targeting cell cycle and metabolic regulators. (4) ceRNA networks integrate lncRNA-miRNA-mRNA interactions to coordinate hyphal growth. These findings provide novel insights into the regulatory framework of filamentous fungal morphogenesis and lay a theoretical foundation for RNA interference-based antifungal strategies.

## ﻿Additional information

### Use of AI

No use of AI was reported.

### Adherence to national and international regulations

All necessary permits were obtained.

### Author ORCIDs

Wudian Xiao https://orcid.org/0000-0002-2718-4298

### Data availability

Data from this study have been deposited in public repositories as follows: Genomic raw reads and scaffolding data have been deposited to the Genome Sequence Archive (GSA) of the China National Center for Bioinformation (CNCB) under accession CRA028745. The final assembled and annotated genome is available from the CNCB Genome Warehouse (GWH) under accession GWHGQOF00000000.1. All transcriptomic sequencing data have been submitted to the NCBI Sequence Read Archive (SRA) under accessions SRR26663347–SRR26663352 (mRNA/lncRNA) and SRR26416206–SRR26416211 (miRNA).

## Supplementary materials

Supplementary material 1 Supplementary figures S1–S5 Data type zip Explanation note **figure S1.***T. mentagrophytes* observed by optical microscopy during germination. (A) 0 h, (B) 18 h. Scale bar: 20 μm. **figure S2.** Assessment of Sequencing Saturation across all samples. (A) Sequencing saturation distribution of sample S1. (B) Sequencing saturation distribution of sample S2. (C) Sequencing saturation distribution of sample S3. (D) Sequencing saturation distribution of sample H1. (E) Sequencing saturation distribution of sample H2. (F) Sequencing saturation distribution of sample H3. **figure S3.** Classification of clean reads based on hierarchical genomic mapping. The mapping status of clean reads hierarchically aligned to non-coding RNA in GenBank (A), genome (B), exon regions (C), and repeat sequence (D). **figure S4.** Nucleotide bias analysis of known and novel miRNAs in hyphae and spore samples. First nuleotide bias of known miRNAs of hyphe (A) and spore (B). First nuleotide bias of novel miRNAs of hyphe (C) and spore (D). Nucleotide bias at each position of known miRNAs of hyphe (E) and spore (F). Nucleotide bias at each position of novel miRNAs of hyphe (G) and spore (H). **figure S5.** Analysis of small RNA composition and miRNA expression profile. (A) Comprehensive annotation of small RNA tags. (B) miRNA expression violin plot. *p* = 0.0416*. https://binary.pensoft.net/file/1457787This dataset is made available under the Open Database License (http://opendatacommons.org/licenses/odbl/1.0/). The Open Database License (ODbL) is a license agreement intended to allow users to freely share, modify, and use this Dataset while maintaining this same freedom for others, provided that the original source and author(s) are credited.Wudian Xiao, Zhaodan Wu, Jia Zhang, Jun Wan, Ruihuan Zhang, Xinyi Xiang, Yang Yu, Lu Fu, Kui Yang, Yang Chen, Ziyao Xiao, Ziyu Wang, Lvqin He, Jingcan You, Chunxiang Zhang

Supplementary material 2 Supplementary tables S1–S13 Data type zip Explanation note **table S1.** Summary of sequencing libraries for *T. mentagrophytes* spore and hyphal samples. **table S2.** Identification and differential expression analysis of mRNAs in *T. mentagrophytes* spore and hyphal samples. **table S3.** Functional enrichment analysis of differentially expressed mRNAs. **table S4.** Gene set enrichment analysis of differentially expressed mRNAs. **table S5.** Analysis of the protein-protein interaction network. **table S6.** Identification and differential expression analysis of lncRNAs. **table S7.** List of *antisense* lncRNA-mRNA regulatory pairs and their functional enrichment. **table S8.** List of *cis* lncRNA-mRNA regulatory pairs and their functional enrichment. **table S9.** List of *trans* lncRNA-mRNA regulatory pairs and their functional enrichment. **table S10.** Identification and differential expression analysis of miRNAs. **table S11.** Functional enrichment analysis of miRNA target genes. **table S12.** CeRNA network pairs and functional enrichment analysis of ceRNA-related mRNAs. **table S13.** Primer sequences and qRT-PCR validation results. https://binary.pensoft.net/file/1457788This dataset is made available under the Open Database License (http://opendatacommons.org/licenses/odbl/1.0/). The Open Database License (ODbL) is a license agreement intended to allow users to freely share, modify, and use this Dataset while maintaining this same freedom for others, provided that the original source and author(s) are credited.Wudian Xiao, Zhaodan Wu, Jia Zhang, Jun Wan, Ruihuan Zhang, Xinyi Xiang, Yang Yu, Lu Fu, Kui Yang, Yang Chen, Ziyao Xiao, Ziyu Wang, Lvqin He, Jingcan You, Chunxiang Zhang

## References

[B1] Bitencourt TA Neves-da-Rocha J Martins MP (2021) StuA-Regulated Processes in the Dermatophyte Trichophyton rubrum: Transcription Profile, Cell-Cell Adhesion, and Immunomodulation. Frontiers in Cellular and Infection Microbiology 11: 643659. 10.3389/fcimb.2021.643659 PMC8218993 34169004

[B2] Bleichrodt RJ Foster P Howell G (2020) Cell wall composition heterogeneity between single cells in *Aspergillus fumigatus* leads to heterogeneous behavior during antifungal treatment and phagocytosis. mBio 11(3): e03015-19. 10.1128/mBio.03015-19 PMC7218287 32398317

[B3] Bowman SM Piwowar A Al Dabbous M (2006) Mutational analysis of the glycosylphosphatidylinositol (GPI) anchor pathway demonstrates that GPI-anchored proteins are required for cell wall biogenesis and normal hyphal growth in *Neurospora crassa*. Eukaryotic Cell 5(3): 587–600. 10.1128/EC.5.3.587-600.2006 16524913 PMC1398062

[B4] Browning H Hackney DD (2005) The EB1 homolog Mal3 stimulates the ATPase of the kinesin Tea2 by recruiting it to the microtubule. The Journal of Biological Chemistry 280(13): 12299–12304. 10.1074/jbc.M413620200 15665379

[B5] Cesana M Cacchiarelli D Legnini I (2011) A long noncoding RNA controls muscle differentiation by functioning as a competing endogenous RNA. Cell 147(2): 358–369. 10.1016/j.cell.2011.09.028 22000014 PMC3234495

[B6] Chardwiriyapreecha S Shimazu M Morita T (2008) Identification of the *fnx1*^+^ and *fnx2*^+^ genes for vacuolar amino acid transporters in *Schizosaccharomyces pombe*. FEBS Letters 582(15): 2225–2230. 10.1016/j.febslet.2008.05.017 18503766

[B7] Chen LL Kim VN (2024) Small and long non-coding RNAs: Past, present, and future. Cell 187(23): 6451–6485. 10.1016/j.cell.2024.10.024 39547208

[B8] Chen H Wang Z Wang Z (2016) Improving methionine and ATP availability by MET6 and SAM2 co-expression combined with sodium citrate feeding enhanced SAM accumulation in Saccharomyces cerevisiae. World Journal of Microbiology & Biotechnology 32(4): 56. 10.1007/s11274-016-2010-y 26925618

[B9] Chen S Zhou Y Chen Y (2018) fastp: An ultra-fast all-in-one FASTQ preprocessor. Bioinformatics (Oxford, England) 34(17): i884–i890. 10.1093/bioinformatics/bty560 PMC6129281 30423086

[B10] Chermette R Ferreiro L Guillot J (2008) Dermatophytoses in animals. Mycopathologia 166(5–6): 385–405. 10.1007/s11046-008-9102-7 18478363

[B11] Cieśla M Mierzejewska J Adamczyk M (2014) Fructose bisphosphate aldolase is involved in the control of RNA polymerase III-directed transcription. Biochimica et Biophysica Acta. Molecular Cell Research 1843(6): 1103–1110. 10.1016/j.bbamcr.2014.02.007 24576411

[B12] Cloutier SC Wang S Ma WK (2016) Regulated formation of lncRNA-DNA hybrids enables faster transcriptional induction and environmental adaptation. Molecular Cell 61(3): 393–404. 10.1016/j.molcel.2015.12.024 26833086 PMC4744127

[B13] Cumsky MG McEwen JE Ko C (1983) Nuclear genes for mitochondrial proteins. Identification and isolation of a structural gene for subunit V of yeast cytochrome c oxidase. The Journal of Biological Chemistry 258(22): 13418–13421. 10.1016/S0021-9258(17)43929-9 6315696

[B14] de Curcio JS Oliveira LN Batista MP (2021) MiRNAs regulate iron homeostasis in *Paracoccidioides brasiliensis*. Microbes and Infection 23(2–3): 104772. 10.1016/j.micinf.2020.10.008 33157279

[B15] Duek L Kaufman G Ulman Y (2004) The pathogenesis of dermatophyte infections in human skin sections. The Journal of Infection 48(2): 175–180. 10.1016/j.jinf.2003.09.008 14720494

[B16] Ebert A Monod M Salamin K (2020) Alarming India-wide phenomenon of antifungal resistance in dermatophytes: A multicentre study. Mycoses 63(7): 717–728. 10.1111/myc.13091 32301159

[B17] Elavarashi E Kindo AJ Rangarajan S (2017) Enzymatic and non-enzymatic virulence activities of dermatophytes on solid media. Journal of Clinical and Diagnostic Research 11: Dc23–Dc25. 10.7860/JCDR/2017/23147.9410 PMC5376860 28384862

[B18] Elowe S (2011) Bub1 and BubR1: At the interface between chromosome attachment and the spindle checkpoint. Molecular and Cellular Biology 31(15): 3085–3093. 10.1128/MCB.05326-11 21628528 PMC3147602

[B19] Fang J Hogan GJ Liang G (2007) The *Saccharomyces cerevisiae* histone demethylase Jhd1 fine-tunes the distribution of H3K36me2. Molecular and Cellular Biology 27(13): 5055–5065. 10.1128/MCB.00127-07 17470555 PMC1951470

[B20] Fernandez J Wilson RA (2014) Cells in cells: Morphogenetic and metabolic strategies conditioning rice infection by the blast fungus *Magnaporthe oryzae*. Protoplasma 251(1): 37–47. 10.1007/s00709-013-0541-8 23990109

[B21] Fernandez J Marroquin-Guzman M Wilson RA (2014) Evidence for a transketolase-mediated metabolic checkpoint governing biotrophic growth in rice cells by the blast fungus Magnaporthe oryzae. PLOS Pathogens 10(9): e1004354. 10.1371/journal.ppat.1004354 PMC4154871 25188286

[B22] Friend JE Sayyad WA Arasada R (2018) Fission yeast Myo2: Molecular organization and diffusion in the cytoplasm. Cytoskeleton 75(4): 164–173. 10.1002/cm.21425 29205883 PMC5899921

[B23] Galvão-Rocha FM Rocha CHL Martins MP (2023) The antidepressant sertraline affects cell signaling and metabolism in *Trichophyton rubrum*. Journal of Fungi (Basel, Switzerland) 9(2): 275. 10.3390/jof9020275 36836389 PMC9961077

[B24] Govindan B Bowser R Novick P (1995) The role of Myo2, a yeast class V myosin, in vesicular transport. The Journal of Cell Biology 128(6): 1055–1068. 10.1083/jcb.128.6.1055 7896871 PMC2120422

[B25] Harris SD (2005) Morphogenesis in germinating *Fusarium graminearum* macroconidia. Mycologia 97(4): 880–887. 10.1080/15572536.2006.11832779 16457357

[B26] Hashida-Okado T Ogawa A Endo M (1996) AUR1, a novel gene conferring aureobasidin resistance on *Saccharomyces cerevisiae*: A study of defective morphologies in Aur1p-depleted cells. Molecular & General Genetics 251(2): 236–244. 10.1007/BF02172923 8668135

[B27] Hiraishi H Miyake T Ono B (2008) Transcriptional regulation of *Saccharomyces cerevisiae* CYS3 encoding cystathionine gamma-lyase. Current Genetics 53(4): 225–234. 10.1007/s00294-008-0181-2 18317767 PMC2668581

[B28] Hirozumi K Nakajima H Machida M (1999) Cloning and characterization of a gene (arpA) from *Aspergillus oryzae* encoding an actin-related protein required for normal nuclear distribution and morphology of conidiophores. Molecular & General Genetics 262(4–5): 758–767. 10.1007/s004380051138 10628858

[B29] Hombach S Kretz M (2016) Non-coding RNAs: Classification, biology and functioning. Advances in Experimental Medicine and Biology 937: 3–17. 10.1007/978-3-319-42059-2_1 27573892

[B30] Horng JS Linz JE Pestka JJ (1989) Cloning and characterization of the trpC gene from an aflatoxigenic strain of *Aspergillus parasiticus*. Applied and Environmental Microbiology 55(10): 2561–2568. 10.1128/aem.55.10.2561-2568.1989 2690735 PMC203122

[B31] Hrdlickova R Toloue M Tian B (2017) RNA-Seq methods for transcriptome analysis. Wiley Interdisciplinary Reviews. RNA 8(1): e1364. 10.1002/wrna.1364 PMC5717752 27198714

[B32] Huang P Yu X Liu H (2024) Regulation of TRI5 expression and deoxynivalenol biosynthesis by a long non-coding RNA in *Fusarium graminearum*. Nature Communications 15(1): 1216. 10.1038/s41467-024-45502-w PMC10853542 38332031

[B33] Iwakawa HO Tomari Y (2022) Life of RISC: Formation, action, and degradation of RNA-induced silencing complex. Molecular Cell 82(1): 30–43. 10.1016/j.molcel.2021.11.026 34942118

[B34] Karnik R Zhang B Waghmare S (2015) Binding of SEC11 indicates its role in SNARE recycling after vesicle fusion and identifies two pathways for vesicular traffic to the plasma membrane. The Plant Cell 27(3): 675–694. 10.1105/tpc.114.134429 25747882 PMC4558655

[B35] Karuppiah V Zhang C Liu T (2023) Transcriptome analysis of *T. asperellum* GDFS 1009 revealed the role of MUP1 gene on the methionine-based induction of morphogenesis and biological control activity. Journal of Fungi (Basel, Switzerland) 9(2): 215. 10.3390/jof9020215 36836329 PMC9963050

[B36] Kim D Langmead B Salzberg SL (2015) HISAT: A fast spliced aligner with low memory requirements. Nature Methods 12(4): 357–360. 10.1038/nmeth.3317 25751142 PMC4655817

[B37] Kim GL Lee S Luong TT (2017) Effect of decreased BCAA synthesis through disruption of ilvC gene on the virulence of *Streptococcus pneumoniae*. Archives of Pharmacal Research 40(8): 921–932. 10.1007/s12272-017-0931-0 28735462

[B38] Kong L Zhang Y Ye ZQ (2007) CPC: Assess the protein-coding potential of transcripts using sequence features and support vector machine. Nucleic Acids Research 35(suppl_2): W345–W349. 10.1093/nar/gkm391 PMC1933232 17631615

[B39] Lai T Yu Q Pan J (2023) The identification and comparative analysis of non-coding RNAs in spores and mycelia of *Penicillium expansum*. Journal of Fungi (Basel, Switzerland) 9(10): 999. 10.3390/jof9100999 37888255 PMC10607695

[B40] Langmead B Salzberg SL (2012) Fast gapped-read alignment with Bowtie 2. Nature Methods 9(4): 357–359. 10.1038/nmeth.1923 22388286 PMC3322381

[B41] Lau AYT Xie Y Cheung MK (2020) Genome-wide mRNA and miRNA analysis in the early stages of germ tube outgrowth in *Coprinopsis cinerea*. Fungal Genetics and Biology 142: 103416. 10.1016/j.fgb.2020.103416 32522620

[B42] Li B Dewey CN (2011) RSEM: Accurate transcript quantification from RNA-Seq data with or without a reference genome. BMC Bioinformatics 12(1): 323. 10.1186/1471-2105-12-323 21816040 PMC3163565

[B43] Li P Ruan X Yang L (2015) A liver-enriched long non-coding RNA, lncLSTR, regulates systemic lipid metabolism in mice. Cell Metabolism 21(3): 455–467. 10.1016/j.cmet.2015.02.004 25738460 PMC4350020

[B44] Li Y Syed J Sugiyama H (2016) RNA-DNA triplex formation by long noncoding RNAs. Cell Chemical Biology 23(11): 1325–1333. 10.1016/j.chembiol.2016.09.011 27773629

[B45] Li Y Xian H Xu Y (2021) Fine tuning the glycolytic flux ratio of EP-bifido pathway for mevalonate production by enhancing glucose-6-phosphate dehydrogenase (Zwf) and CRISPRi suppressing 6-phosphofructose kinase (PfkA) in *Escherichia coli*. Microbial Cell Factories 20(1): 32. 10.1186/s12934-021-01526-1 33531004 PMC7852082

[B46] Liu T Zhang Q Wang L (2007) The use of global transcriptional analysis to reveal the biological and cellular events involved in distinct development phases of *Trichophyton rubrum* conidial germination. BMC Genomics 8(1): 100. 10.1186/1471-2164-8-100 17428342 PMC1871584

[B47] Liu H Zhang S Ma J (2015) Two Cdc2 kinase genes with distinct functions in vegetative and infectious hyphae in *Fusarium graminearum*. PLOS Pathogens 11(6): e1004913. 10.1371/journal.ppat.1004913 PMC4470668 26083253

[B48] Liu B Xiang W Liu J (2021) The regulatory role of antisense lncRNAs in cancer. Cancer Cell International 21(1): 459. 10.1186/s12935-021-02168-4 34461912 PMC8404292

[B49] Liu H Li D Sun L (2022a) Interaction of lncRNA MIR100HG with hnRNPA2B1 facilitates m(6)A-dependent stabilization of TCF7L2 mRNA and colorectal cancer progression. Molecular Cancer 21(1): 74. 10.1186/s12943-022-01555-3 35279145 PMC8917698

[B50] Liu N Xu Y Li Q (2022b) A lncRNA fine-tunes salicylic acid biosynthesis to balance plant immunity and growth. Cell Host & Microbe 30(8): 1124–1138. 10.1016/j.chom.2022.07.001 35908550

[B51] Livak KJ Schmittgen TD (2001) Analysis of relative gene expression data using real-time quantitative PCR and the 2(-Delta Delta C(T)). Methods: A Companion to Methods in Enzymology 25(4): 402–408. 10.1006/meth.2001.1262 11846609

[B52] Love MI Huber W Anders S (2014) Moderated estimation of fold change and dispersion for RNA-seq data with DESeq2. Genome Biology 15(12): 550. 10.1186/s13059-014-0550-8 25516281 PMC4302049

[B53] Lu X Xu Q Tong Y (2022) Long non-coding RNA EVADR induced by *Fusobacterium nucleatum* infection promotes colorectal cancer metastasis. Cell Reports 40(3): 111127. 10.1016/j.celrep.2022.111127 35858553

[B54] Luzzani C Cardillo SB Bermúdez Moretti M (2007) New insights into the regulation of the *Saccharomyces cerevisiae* UGA4 gene: Two parallel pathways participate in carbon-regulated transcription. Microbiology (Reading, England) 153(11): 3677–3684. 10.1099/mic.0.2007/010231-0 17975075

[B55] Mackowiak SD (2011) Identification of novel and known miRNAs in deep-sequencing data with miRDeep2. Curr Protoc Bioinformatics Chapter 12: 12.10.11–12.10.15. 10.1002/0471250953.bi1210s36 22161567

[B56] Maddi A Bowman SM Free SJ (2009) Trifluoromethanesulfonic acid-based proteomic analysis of cell wall and secreted proteins of the ascomycetous fungi *Neurospora crassa* and *Candida albicans*. Fungal Genetics and Biology 46(10): 768–781. 10.1016/j.fgb.2009.06.005 19555771 PMC2728151

[B57] Masuda H Toda T Miyamoto R (2006) Modulation of Alp4 function in *Schizosaccharomyces pombe* induces novel phenotypes that imply distinct functions for nuclear and cytoplasmic gamma-tubulin complexes. Genes to Cells 11(4): 319–336. 10.1111/j.1365-2443.2006.00946.x 16611237

[B58] Masuda H Fong CS Ohtsuki C (2011) Spatiotemporal regulations of Wee1 at the G2/M transition. Molecular Biology of the Cell 22(5): 555–569. 10.1091/mbc.e10-07-0644 21233285 PMC3046054

[B59] Mellado E Dubreucq G Mol P (2003) Cell wall biogenesis in a double chitin synthase mutant (chsG-/chsE-) of *Aspergillus fumigatus*. Fungal Genetics and Biology 38(1): 98–109. 10.1016/S1087-1845(02)00516-9 12553940

[B60] Nahkuri S Paro R (2012) The role of noncoding RNAs in chromatin regulation during differentiation. Wiley Interdisciplinary Reviews. Developmental Biology 1(5): 743–752. 10.1002/wdev.41 23799570

[B61] Novačić A Vučenović I Primig M (2020) Non-coding RNAs as cell wall regulators in *Saccharomyces cerevisiae*. Critical Reviews in Microbiology 46(1): 15–25. 10.1080/1040841X.2020.1715340 31994960

[B62] Oakley BR (2004) Tubulins in *Aspergillus nidulans*. Fungal Genetics and Biology 41(4): 420–427. 10.1016/j.fgb.2003.11.013 14998525

[B63] Orasch T Dietl AM Shadkchan Y (2019) The leucine biosynthetic pathway is crucial for adaptation to iron starvation and virulence in *Aspergillus fumigatus*. Virulence 10(1): 925–934. 10.1080/21505594.2019.1682760 31694453 PMC6844326

[B64] Osherov N May G (2000) Conidial germination in *Aspergillus nidulans* requires RAS signaling and protein synthesis. Genetics 155(2): 647–656. 10.1093/genetics/155.2.647 10835388 PMC1461097

[B65] Pascon RC Ganous TM Kingsbury JM (2004) Cryptococcus neoformans methionine synthase: Expression analysis and requirement for virulence. Microbiology (Reading, England) 150(9): 3013–3023. 10.1099/mic.0.27235-0 15347759

[B66] Pertea M Pertea GM Antonescu CM (2015) StringTie enables improved reconstruction of a transcriptome from RNA-seq reads. Nature Biotechnology 33(3): 290–295. 10.1038/nbt.3122 PMC4643835 25690850

[B67] Rampitsch C Tinker NA Subramaniam R (2012) Phosphoproteome profile of *Fusarium graminearum* grown in vitro under nonlimiting conditions. Proteomics 12(7): 1002–1005. 10.1002/pmic.201100065 22522806

[B68] Rödel C Jupitz T Schmidt H (1997) Complementation of the DNA repair-deficient swi10 mutant of fission yeast by the human ERCC1 gene. Nucleic Acids Research 25(14): 2823–2827. 10.1093/nar/25.14.2823 9207030 PMC146808

[B69] Saçar Demirci MD (2020) Computational prediction of microRNAs in *Histoplasma capsulatum*. Microbial Pathogenesis 148: 104433. 10.1016/j.micpath.2020.104433 32858119

[B70] Salmena L Poliseno L Tay Y (2011) A ceRNA hypothesis: The Rosetta Stone of a hidden RNA language? Cell 146(3): 353–358. 10.1016/j.cell.2011.07.014 PMC3235919 21802130

[B71] Shannon P Markiel A Ozier O (2003) Cytoscape: A software environment for integrated models of biomolecular interaction networks. Genome Research 13(11): 2498–2504. 10.1101/gr.1239303 14597658 PMC403769

[B72] Shuman S (2020) Transcriptional interference at tandem lncRNA and protein-coding genes: An emerging theme in regulation of cellular nutrient homeostasis. Nucleic Acids Research 48(15): 8243–8254. 10.1093/nar/gkaa630 32720681 PMC7470944

[B73] Sousa S McLaughlin MM Pereira SA (2002) The ARO4 gene of *Candida albicans* encodes a tyrosine-sensitive DAHP synthase: Evolution, functional conservation and phenotype of Aro3p-, Aro4p-deficient mutants. Microbiology (Reading, England) 148(5): 1291–1303. 10.1099/00221287-148-5-1291 11988503

[B74] Steyer JT Todd RB (2023) Branched-chain amino acid biosynthesis in fungi. Essays in Biochemistry 67(5): 865–876. 10.1042/EBC20230003 37455545

[B75] Steyer JT Downes DJ Hunter CC (2021) Duplication and functional divergence of branched-chain amino acid biosynthesis genes in *Aspergillus nidulans*. mBio 12(3): e0076821. 10.1128/mBio.00768-21 PMC8262921 34154419

[B76] Subramanian A Tamayo P Mootha VK (2005) Gene set enrichment analysis: A knowledge-based approach for interpreting genome-wide expression profiles. Proceedings of the National Academy of Sciences of the United States of America 102(43): 15545–15550. 10.1073/pnas.0506580102 16199517 PMC1239896

[B77] Sun J Li H Sun X (2012) Trisporic acid stimulates gene transcription of terpenoid biosynthesis in *Blakeslea trispora*. Process Biochemistry (Barking, London, England) 47(12): 1889–1893. 10.1016/j.procbio.2012.06.017

[B78] Sun L Luo H Bu D (2013) Utilizing sequence intrinsic composition to classify protein-coding and long non-coding transcripts. Nucleic Acids Research 41(17): e166. 10.1093/nar/gkt646 PMC3783192 23892401

[B79] Szklarczyk D Franceschini A Wyder S (2015) STRING v10: Protein-protein interaction networks, integrated over the tree of life. Nucleic Acids Research 43(D1): D447–D452. 10.1093/nar/gku1003 PMC4383874 25352553

[B80] Tafer H Hofacker IL (2008) RNAplex: A fast tool for RNA-RNA interaction search. Bioinformatics (Oxford, England) 24(22): 2657–2663. 10.1093/bioinformatics/btn193 18434344

[B81] Trapnell C Williams BA Pertea G (2010) Transcript assembly and quantification by RNA-Seq reveals unannotated transcripts and isoform switching during cell differentiation. Nature Biotechnology 28(5): 511–515. 10.1038/nbt.1621 PMC3146043 20436464

[B82] Wang L Xu X Yang J (2018) Integrated microRNA and mRNA analysis in the pathogenic filamentous fungus *Trichophyton rubrum*. BMC Genomics 19(1): 933. 10.1186/s12864-018-5316-3 30547762 PMC6295003

[B83] Wang Z Yu Z He L (2022) Comprehensive analysis of long non-coding RNA expression profiles in *Trichophyton mentagrophytes*-infected keratinocytes. Microbial Pathogenesis 167: 105565. 10.1016/j.micpath.2022.105565 35523366

[B84] Weitzman I Summerbell RC (1995) The dermatophytes. Clinical Microbiology Reviews 8(2): 240–259. 10.1128/CMR.8.2.240 7621400 PMC172857

[B85] Westfall PJ Momany M (2002) *Aspergillus nidulans* septin AspB plays pre- and postmitotic roles in septum, branch, and conidiophore development. Molecular Biology of the Cell 13(1): 110–118. 10.1091/mbc.01-06-0312 11809826 PMC65076

[B86] Wu H Cen Y Lu Y (2024) Role of chitin synthases CHS1 and CHS2 in biosynthesis of the cyst wall of *Cryptocaryon irritans*. International Journal of Biological Macromolecules 280: 136143. 10.1016/j.ijbiomac.2024.136143 39357726

[B87] Wucher V Legeai F Hédan B (2017) FEELnc: A tool for long non-coding RNA annotation and its application to the dog transcriptome. Nucleic Acids Research 45: e57. 10.1093/nar/gkw1306 PMC5416892 28053114

[B88] Yan P Luo S Lu JY (2017) *Cis*- and *trans*-acting lncRNAs in pluripotency and reprogramming. Current Opinion in Genetics & Development 46: 170–178. 10.1016/j.gde.2017.07.009 28843809

[B89] Yoon JH Abdelmohsen K Gorospe M (2013) Posttranscriptional gene regulation by long noncoding RNA. Journal of Molecular Biology 425(19): 3723–3730. 10.1016/j.jmb.2012.11.024 23178169 PMC3594629

[B90] Zang F Wang Z Yang Y (2024) Responses of keratinocytes to *Trichophyton mentagrophytes* infection based on whole transcriptome analysis. Mycoses 67(3): e13713. 10.1111/myc.13713 38483066

[B91] Zhang J Zeng L Wu Z (2023) Genome-wide identification and functional analysis of circRNAs in *Trichophyton mentagrophytes* spores and hyphae. Microbial Pathogenesis 176: 106003. 10.1016/j.micpath.2023.106003 36702368

