# Supplementary figures and images for: ﻿Stage-specific RNA regulomes of Trichophyton mentagrophytes: mRNA-lncRNA-miRNA interplay in spore-hypha transition
Source: IMA Fungus. 2025 Nov 5;16:e166433. doi: 10.3897/imafungus.16.166433 (PMC12612851; doi:10.3897/imafungus.16.166433)

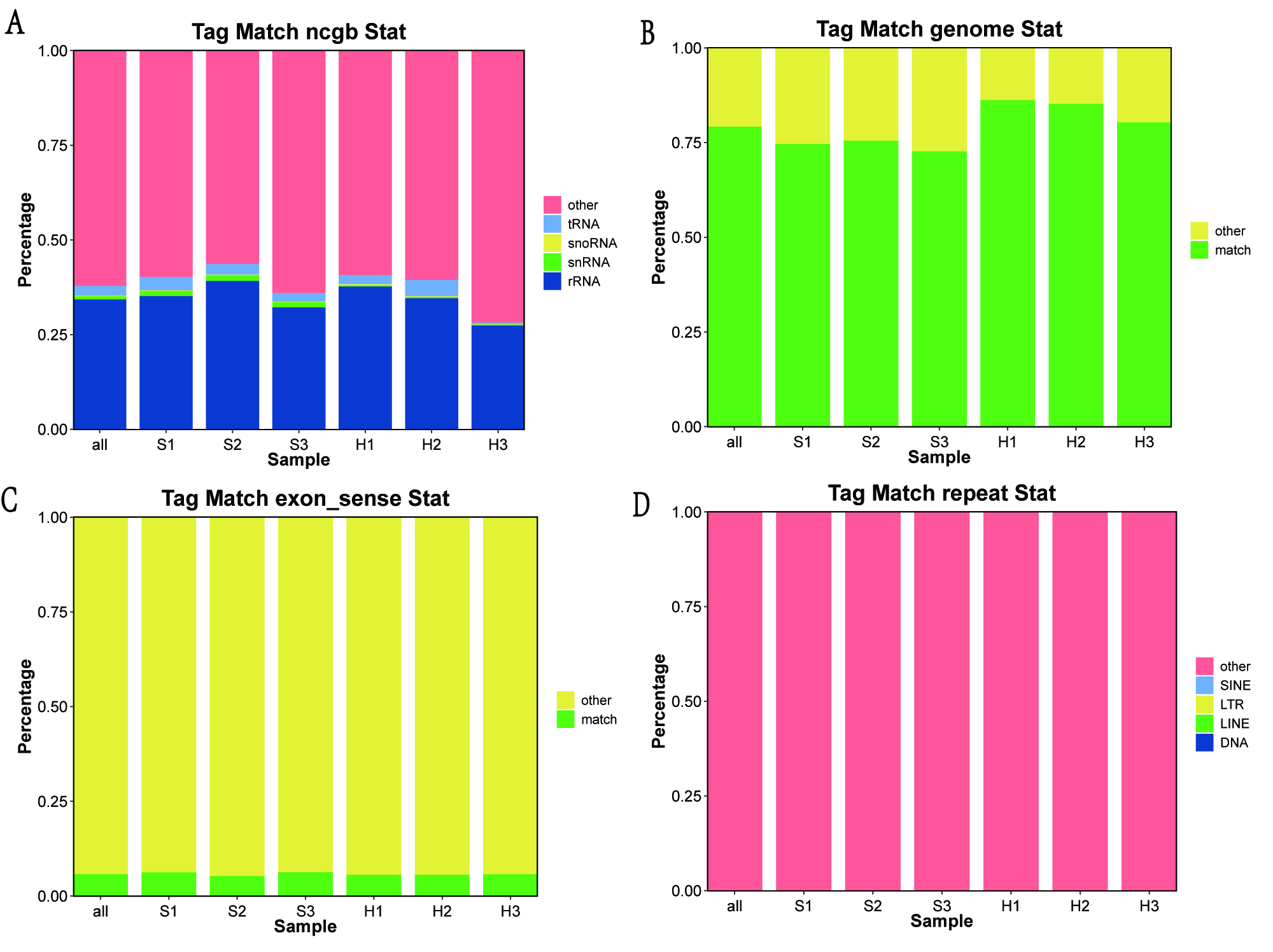

Supplement: Supplementary material 1 — Supplementary figures S1–S5 [file imafungus-16-e166433-s001.zip › Fig S3.tif]

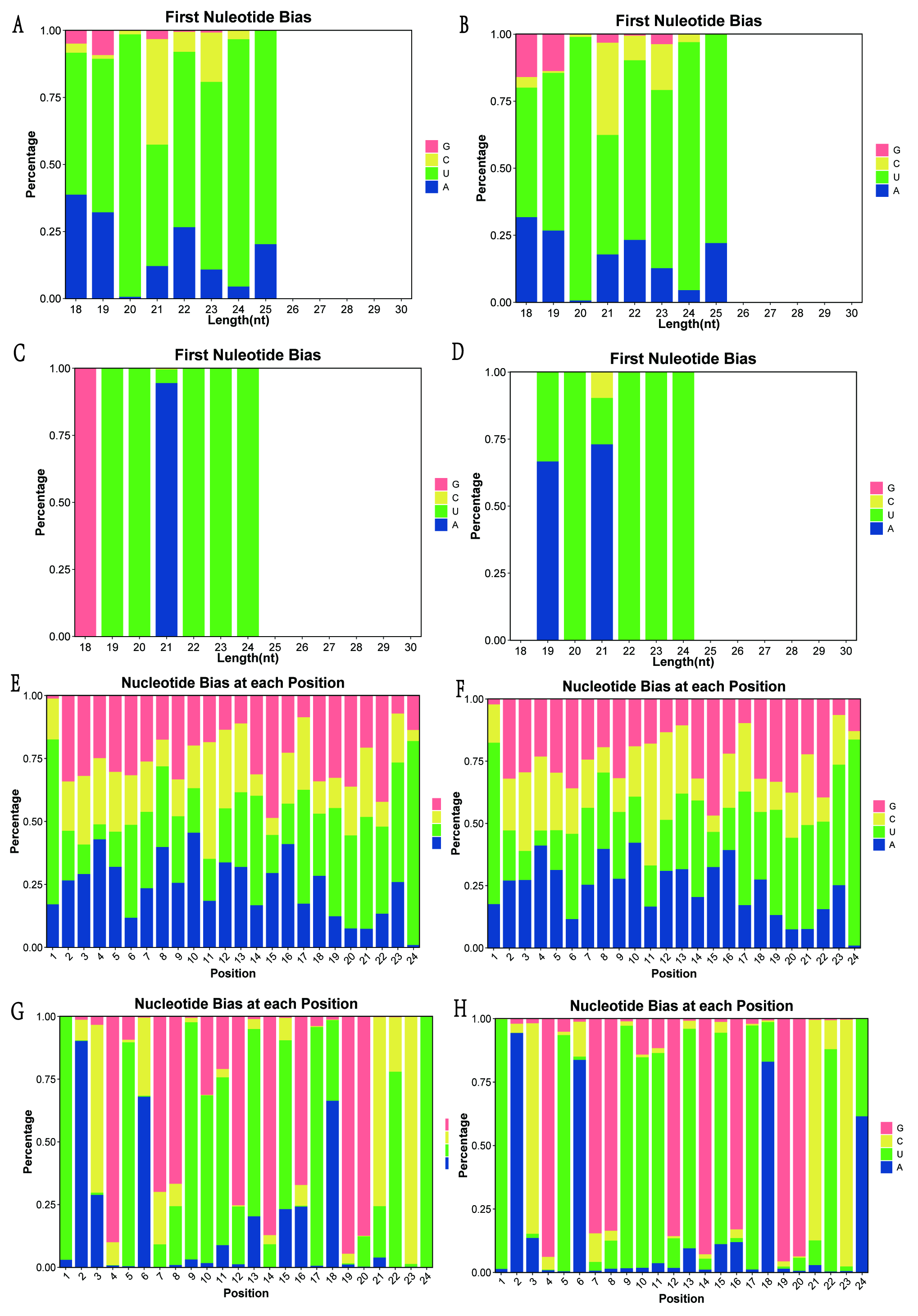

Supplement: Supplementary material 1 — Supplementary figures S1–S5 [file imafungus-16-e166433-s001.zip › Fig S4.tif]

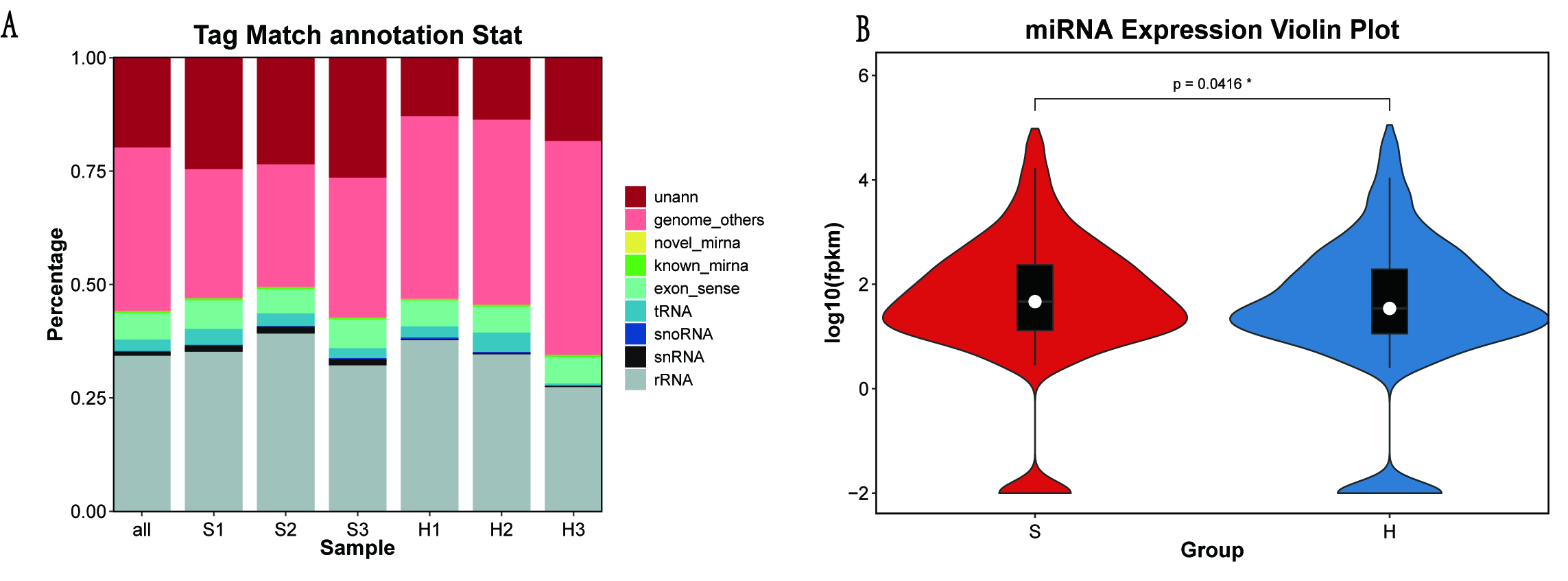

Supplement: Supplementary material 1 — Supplementary figures S1–S5 [file imafungus-16-e166433-s001.zip › 166433_0R-2-A_revised_Figure_S5.tif]

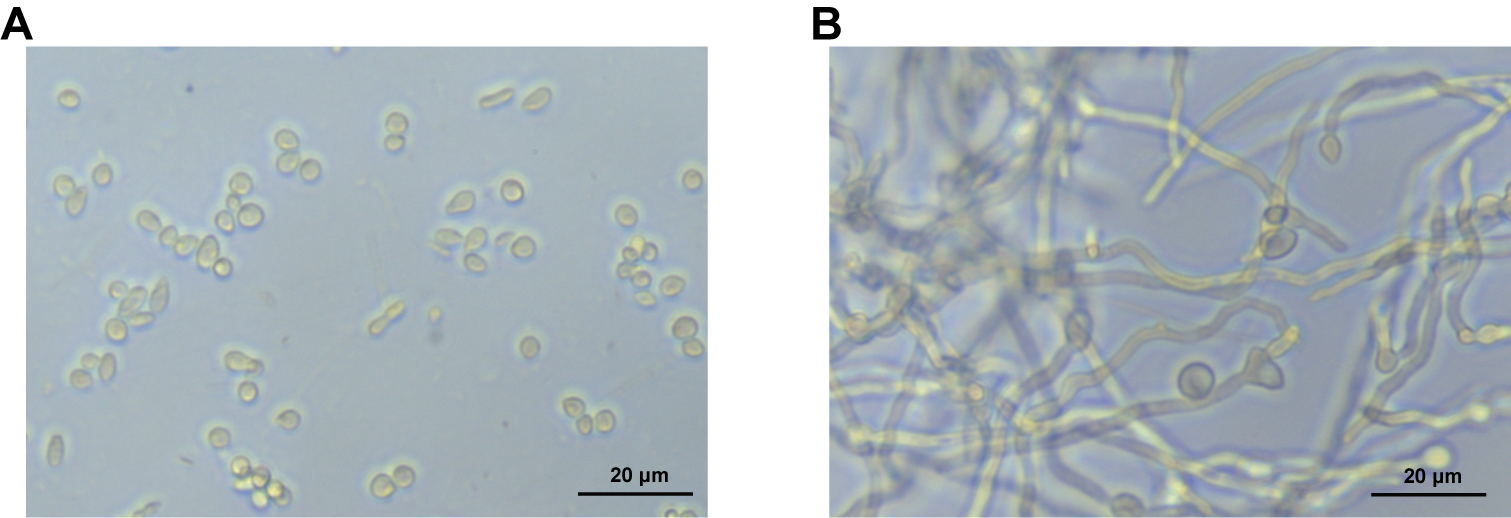

Supplement: Supplementary material 1 — Supplementary figures S1–S5 [file imafungus-16-e166433-s001.zip › Fig S1.tif]

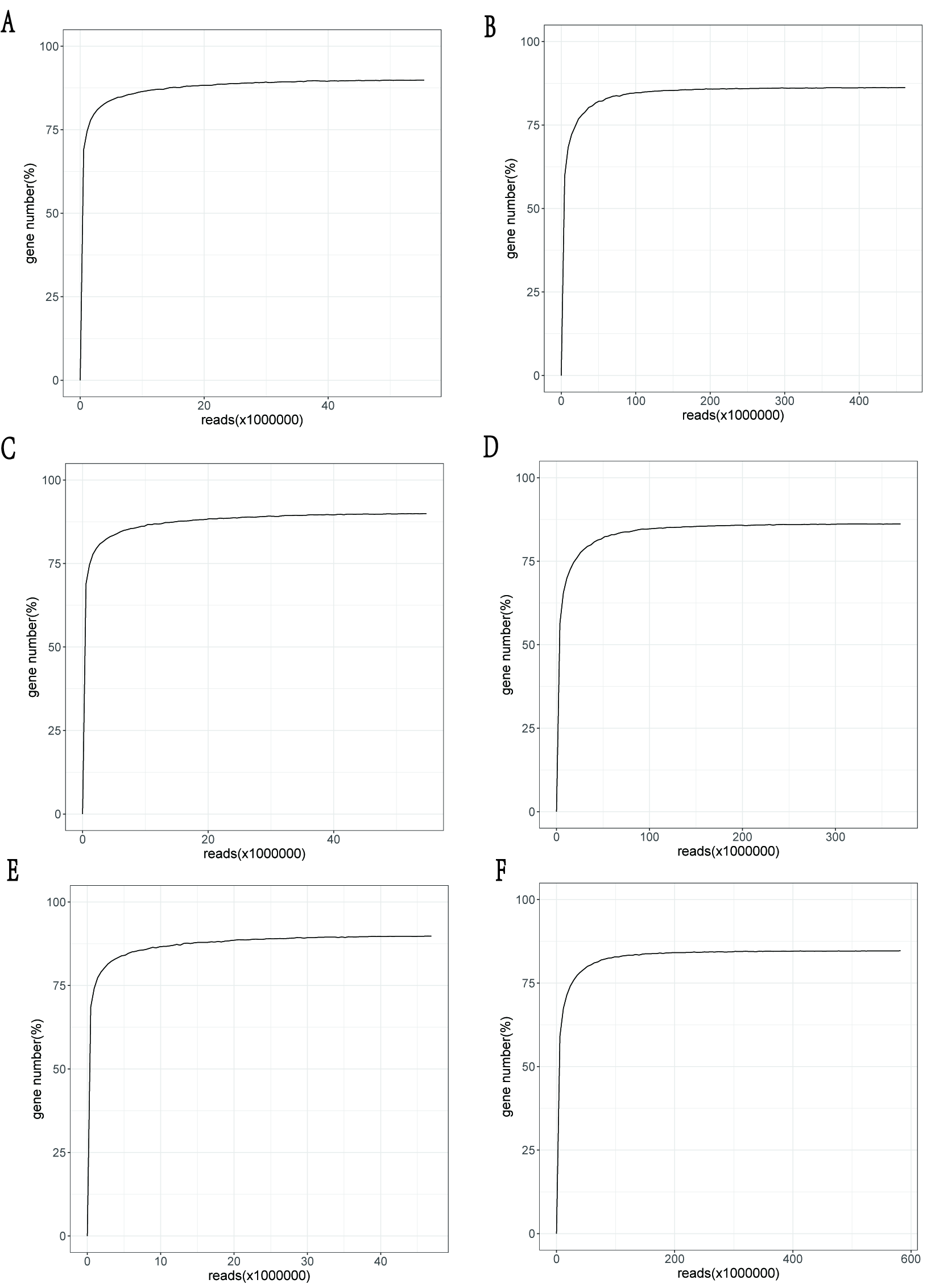

Supplement: Supplementary material 1 — Supplementary figures S1–S5 [file imafungus-16-e166433-s001.zip › Fig S2.tif]
